# ITGA5 as a Dual Regulator of Epithelial‐Mesenchymal Transition and Epithelial Cell Anoikis Resistance: Functional Validation and Drug Prediction

**DOI:** 10.1111/cpr.70190

**Published:** 2026-03-02

**Authors:** Ting Wang, Ling Rao, Xiaofang Li, Miaofen Zhang, Huiting Huang, Zhiyan Luo, Gang Liao, Yong Jiang, Shaofeng Zhan, Qiong Liu, Xiufang Huang

**Affiliations:** ^1^ The First Affiliated Hospital of Guangzhou University of Chinese Medicine Guangzhou China; ^2^ Guangdong Engineering Technology Research Center of Commercialization of Lingnan Special Medical Institution Preparations Guangzhou China; ^3^ Lingnan Medical Research Centre of Guangzhou University of Chinese Medicine Guangzhou China; ^4^ Shenzhen Hospital of Integrated Traditional Chinese and Western Medicine Shenzhen China; ^5^ Guangdong Clinical Research Academy of Chinese Medicine Guangzhou China; ^6^ Guangdong Engineering Research Center of Comercialization of Medical Institution Preparations and Traditional Chinese Medicines Guangzhou China; ^7^ State Key Laboratory of Traditional Chinese Medicine Guangzhou China

**Keywords:** anoikis resistence, epithelial‐mesenchymal transition, ITGA5, pro DIA quantitative proteomics

## Abstract

Airway remodelling is a major contributor to persistent airflow limitation and irreversible lung function impairment in asthma, with epithelial‐mesenchymal transition (EMT) serving as a key driver. However, the molecular mechanisms controlling EMT in asthma epithelium remain incompletely elucidated. This study reported that integrin α5 (ITGA5) was markedly upregulated in asthma patients, house dust mite (HDM)‐sensitised asthma mice, and transforming growth factor beta 1 (TGF‐*β*1)‐induced in vitro EMT models. Elevated ITGA5 expression correlated positively with reduced lung function, asthma severity and higher levels of EMT regulators (Fibronectin, N‐cadherin, Vimentin) and was functionally linked to anoikis resistance. In TGF‐*β*1‐induced bronchial epithelial cells exhibiting anoikis resistance, quantitative proteomics revealed that ITGA5 promoted mesenchymal transition via the phosphoinositide 3‐kinase (PI3K)/protein kinase B (Akt) pathway and negatively regulated anoikis. ITGA5 directly bound to PI3K in vitro, and ITGA5 knockdown reversed TGF‐*β*1‐induced EMT, inhibited the activation of the PI3K/Akt pro‐survival pathway, and restored anoikis sensitivity. According to molecular docking, molecular dynamics simulation and in vivo and in vitro pharmacological assays, resveratrol (Res) and M200 were found to be potential ITGA5 inhibitors that successfully reduced EMT and anoikis resistance, thereby attenuating airway remodelling in asthma mice and offering promising drug candidates for ITGA5‐targeted therapy.

## Graphical abstract

Proteomics and functional validation reveal ITGA5 as a dual promoter of epithelial‐mesenchymal transition and anoikis resistance in human bronchial epithelium: targeting ITGA5 represents a novel therapeutic strategy for asthma airway remodelling.

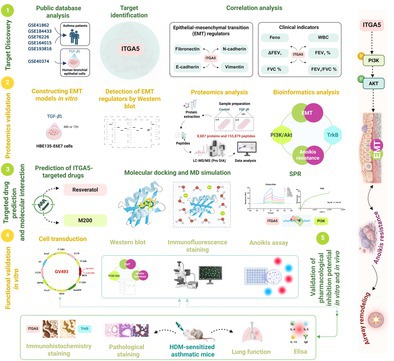

## 1 Introduction

Asthma is characterised by the dysfunction of the airway epithelium, which is crucial to the onset and progression of the condition [1]. Epithelial‐mesenchymal transition (EMT) has emerged as a critical pathological mechanism driving airway remodelling in asthma [2, 3, 4], resulting in structural alterations, impaired lung function and unfavourable clinical outcomes [5]. During EMT, airway epithelial cells lose epithelial hallmarks and acquire a migratory, invasive mesenchymal phenotype, thereby promoting subepithelial fibrosis, airway smooth muscle hyperplasia and mucus hypersecretion [6]. Transforming growth factor beta 1 (TGF‐*β*1) stimulation induces EMT in airway epithelial cells, triggering their transdifferentiation into myofibroblasts and contributing to subepithelial fibrotic remodelling [7, 8]. This process is characterised by decreased expression of epithelial markers (ZO‐1, E‐cadherin, Occludin) and increased expression of mesenchymal markers (Fibronectin, N‐cadherin, Vimentin) [9]. Current therapies for asthma predominantly focus on inflammation, immune modulation and bronchoconstriction, but have limited efficacy in reversing airway remodelling [10, 11, 12, 13, 14]. Consequently, a deeper understanding of the regulatory targets governing airway EMT is essential for elucidating the mechanisms of asthma‐associated remodelling and guiding the development of novel precision therapeutics.

Integrins, a critical family of transmembrane receptors, govern cell adhesion to the extracellular matrix (ECM) and intercellular interactions, playing pivotal roles in the initiation and development of EMT [15, 16]. Among these, integrin *α*5 (ITGA5), a distinct *α*‐subunit of integrins, typically forms a heterodimer with integrin *β*1 to create the *α*5*β*1 integrin complex, which binds fibronectin and connects the intracellular cytoskeleton to the ECM [17, 18]. The high‐affinity interaction between ITGA5 and fibronectin underscores a powerful mechanistic connection between ITGA5, ECM remodelling and EMT [19]. Multiple publications have documented that ITGA5 overexpression directly promotes fibroblast proliferation and fibrotic lesion formation in pulmonary fibrosis [20, 21, 22]. Given these biological functions, we hypothesise that ITGA5 similarly regulates airway epithelial EMT; however, its function in asthma‐associated EMT is still unknown.

Anoikis is a specialised form of programmed cell death that eliminates cells detached from the ECM or nearby cells, thereby maintaining tissue homeostasis and preventing aberrant colonisation [23]. Detached cells develop anoikis resistance when they avoid this death signal and continue to exist without anchoring [24]. Such resistance enables cells to survive and migrate after detachment, providing a survival basis for EMT‐associated changes or the maintenance of a mesenchymal phenotype [25]. Conversely, EMT gives cells more invasiveness and motility, which helps anoikis escape even more, illustrating a complex bidirectional interplay [26]. Indeed, failure of anoikis execution has been observed in fibrotic lung diseases characterised by ECM remodelling [26, 27]. Intriguingly, ITGA5 may indirectly modulate anoikis sensitivity [28] and key EMT behaviours via effects on ECM composition and cell‐ECM interactions [29] due to its roles in adhesion, migration and matrix fibre formation. However, its potential as a predictive marker of anoikis resistance remains uncharacterised.

In this study, we aimed to elucidate the function of ITGA5 in TGF‐*β*1‐induced EMT progression of bronchial epithelial cells and its potential as an anoikis resistance biomarker. Specifically, we sought to validate the functional roles and molecular mechanisms of ITGA5 and screen interventional agents that targeted ITGA5 for pharmacological inhibitory potential assessment both in vitro and in vivo. These findings will provide novel targets and strategies for the development of treatments to stop asthma‐associated airway epithelial EMT.

## 2 Materials and Methods

### 2.1 Collection of Public Data and ITGA5 Expression Analysis

Human gene expression profiles or genome‐wide microarray data of human bronchial epithelial cells were obtained from the Gene Expression Omnibus (GEO) database (https://www.ncbi.nlm.nih.gov/geo/) under accession numbers GSE41862 [30], GSE184433 [31], GSE76226 [32], GSE164015 [33], GSE193816 [34] and GSE40374 [35]. The expression matrices were retrieved using the easyGEO (https://tau.cmmt.ubc.ca/eVITTA/easyGEO/) with statistically significant differential expression of ITGA5 defined as *p* < 0.05.

### 2.2 Pro Data‐Independent Acquisition (DIA) Based Quantitative Proteomic Analysis

Total proteins were extracted by cell lysis and quantified using the bicinchoninic acid (BCA) assay, followed by tryptic digestion to generate peptides. The peptides were desalted, subjected to gradient elution, and separated via ultra‐high‐performance liquid chromatography (UHPLC) on a Vanquish Neo system (Thermo Scientific). Prepared samples were injected into an Orbitrap Astral mass spectrometer (Thermo Scientific) for DIA analysis. Prior to mass spectrometry (MS) injection, indexed retention time (iRT) peptides were spiked into each sample at a 1:20 (v/v) ratio for retention time alignment and internal quality control. MS parameters included: Orbitrap resolution of 240,000; full MS scans (380–980 m/z, automatic gain control (AGC) target: 5, maximum injection time: 5 ms); MS/MS scans (150–2000 m/z, automatic AGC target: 4, maximum injection time: 3 ms, RF lens: 0.4, isolation window: 2 m/z, higher‐energy collisional dissociation (HCD) collision energy: 26%, cycle time: 0.6 s). Finally, all data were processed using DIA‐NN software against a *Homo sapiens* ‐specific UniProt database for peptide identification and DIA‐based protein quantification, ensuring reproducible and precise analysis. The data acquisition workflow was performed by OE Biotech Co. Ltd. (Shanghai, China).

### 2.3 Comprehensive Bioinformatics Analysis

Protein interaction networks centred on ITGA5 were predicted using the STRING database (https://string‐db.org/) and subsequently visualised with the ClueGO plugin in Cytoscape. The DIA protein identification results were visualised via OECloud tools. Principal component analysis (PCA) was conducted based on the expression levels of high‐confidence proteins to assess sample clustering. Differentially expressed proteins (DEPs) were screened with thresholds of *p*‐value < 0.05 and |log2FC| ≥ 1. Subsequently, Gene Ontology (GO) and Kyoto Encyclopaedia of Genes and Genomes (KEGG) enrichment analyses were conducted to explore the biological functions of DEPs. A gene set associated with anoikis resistance was retrieved from GeneCards (https://www.genecards.org/) [36], and the cellular expression patterns of anoikis resistance‐related proteins in human lungs were annotated using the LungMAP Human Lung CellRef [37]. The conjunctive bayesian networks (CBN) plot algorithm was employed to infer pathway regulatory relationships among these proteins. Samples were stratified into ITGA5‐high and ITGA5‐low expression groups based on the median ITGA5 expression, followed by differential analysis using the limma package to identify DEPs for functional enrichment. Finally, potential compounds interacting with ITGA5 were predicted via the CTD (http://ctdbase.org/) [38].

### 2.4 Docking and Molecular Dynamics Simulation (MD)

To assess the pharmacological inhibitory potential of resveratrol (Res) against ITGA5, molecular docking was performed using AutoDock Vina [39], followed by MD simulations with the GROMACS platform to validate complex stability. The small molecule was parameterized with the general amber force field (GAFF) force field, while the protein was described using the AMBER14SB force field coupled with the transferable intermolecular potential 3‐point (TIP3P) water model. The system was subjected to sequential equilibration steps consisting of 100 ps under constant number of particles, volume and temperature (NVT) conditions, followed by 100 ps under constant number of particles, pressure and temperature (NPT) conditions at 298 K, and subsequently a 100 ns production MD simulation of the complex system. Trajectory analyses were conducted via visual molecular dynamics (VMD) and PyMOL for structural visualisation, and binding free energy calculations between ITGA5 and Res were quantitatively evaluated using the gmmpbsa tool based on the molecular mechanics poisson‐boltzmann surface area (MMPBSA) method. To further elucidate the molecular mechanism, protein–protein interactions of ITGA5 with PI3K and TrkB were investigated by global range molecular matching (GRAMM) [40], and detailed docking parameters were analysed via PDBePISA. Normal mode analysis (NMA) implemented on the iMODS server [41] characterised deformation trajectories and dynamic features of protein–protein docking conformations. Structural coordinates of ITGA5, PI3K and tropomyosin receptor kinase B (TrkB) were obtained from the AlphaFold Protein Structure Database (https://alphafold.com/) [42], while ligand files were obtained from the PubChem Compound Database (https://pubchem.ncbi.nlm.nih.gov/) [43].

### 2.5 Cell Culture and Treatment

Human bronchial epithelial cells (HBE135‐E6E7; Jennio Biotech, Guangzhou, China, JNO‐H0016), authenticated by STR profiling, were cultured in keratinocyte medium (KM; ScienCell, US, 2101) supplemented with 1% keratinocyte growth supplement (KGS; ScienCell, US, 2152) and 1% penicillin–streptomycin (P/S; ScienCell, US, 0503) at 37°C in a 5% CO_2_ humidified atmosphere. To induce EMT, cells were exposed to 10 ng/mL recombinant human TGF‐*β*1 (PeproTech, US, 100–21) for 48 h or 72 h following established protocols.

### 2.6 Cell Transduction

ITGA5‐specific shRNA lentiviral system (GeneChem, Shanghai, China) consisted of three independent targeting sequences (sh‐ITGA5#1: GCTCAGATCTTGCTGGACTGT; sh‐ITGA5#2: GCCTGAGCTGTGACTACTTTG; sh‐ITGA5#3: GCAGTGCTATTCCCAGTAAGC) and a negative control sequence (sh‐NC: TTCTCCGAACGTGTCACGT). HBE135‐E6E7 cells in the logarithmic growth phase were transduced with the lentivirus at a multiplicity of infection of 5. At 72 h post‐lentiviral transduction, the fluorescence intensity of green fluorescent protein (GFP) was quantitatively detected using an inverted fluorescence microscope to evaluate the transduction efficiency. Stable ITGA5‐silenced cell lines were selected by treating with 1 μg/mL puromycin medium for 7 days.

### 2.7 Cell Viability Assay

Cell viability was evaluated using the cell counting kit‐8 (CCK‐8) assay. HBE135‐E6E7 cells (2 × 10^4^/well) were seeded in 96‐well plates for 12 h, then treated with Res (0, 0.0625, 0.125, 0.25, 0.5, 1, 2 and 4 μM; Selleck, US, S1396) or M200 (0, 1, 2.5, 5, 10, 20, 40, 80, 100 and 150 nM; MCE, US, HY‐P99333) for 48 h. Cell viability was assessed by 10% CCK‐8 solution (Abbkine, Wuhan, China, BMU106‐CN) incubation (1–4 h) followed by absorbance measurement at 450 nm.

### 2.8 Anoikis Assay

Anoikis was detected following the manufacturer's protocol (Bestbio, Shanghai, China, BB‐4179). Cells (2 × 10^4^ cells/well) were seeded in anti‐adhesion 96‐well plates to induce ECM detachment, then cultured for 48 h. After adding the staining solution, cells were incubated at 37°C for 30–60 min. Using a Leica inverted fluorescence microscope, green Calcein‐AM fluorescence (Ex = 485 nm, Em = 516 nm) indicated anchorage‐independent cells, while red propidium iodide (PI) fluorescence (Ex = 535 nm, Em = 617 nm) marked anoikis‐induced cell death. Anoikis was quantified by determining the Calcein‐AM/PI fluorescence intensity ratio in four randomly acquired microscopic fields.

### 2.9 Western Blotting

Cell or lung samples were lysed in pre‐cooled radioimmunoprecipitation assay (RIPA) lysis buffer (Beyotime, Shanghai, China, P0013C), which contained protease and phosphatase inhibitors to maintain protein integrity. Equal amounts of proteins were separated by sodium dodecyl sulphate‐polyacrylamide gel electrophoresis (SDS‐PAGE) and transferred to a methanol‐activated polyvinylidene fluoride membrane (MilliporeSigma, US, IPVH00010) via wet transfer. After routine blocking, the membrane was incubated overnight at 4°C with Fibronectin (Affinity, AF5335, 1:1000), N‐cadherin (Affinity, AF5239, 1:5000), E‐cadherin (Affinity, AF0131, 1:2000), Vimentin (HuaBio, ET1610‐39, 1:50000), ZO‐1 (HuaBio, HA722797, 1:1000), Occludin (HuaBio, ET1701‐76, 1:2000), phosphorylated phosphatidylinositol 3‐kinase (p‐PI3K) (Affinity, AF3242, 1:1000), PI3K (Affinity, AF6242, 1:1000), phosphorylated protein kinase B (p‐AKT) (Affinity, AF0016, 1:2000), protein kinase B (AKT) (Proteintech, 10176‐2‐AP, 1:6000), ITGA5 (HuaBio, ET1701‐58, 1:2000), TrkB (Affinity, AF6461, 1:1000), Caspase 3 and Cleaved‐caspase 3 (Proteintech, 19677‐1‐AP, 1:1000), Caspase 9 and Cleaved‐caspase 9 (Proteintech, 103807‐1‐AP, 1:600), x‐linked inhibitor of apoptosis protein (XIAP) (Selleck, F0515, 1:1000), myeloid cell leukaemia 1 (MCL1) (Selleck, F0024, 1:1000), glyceraldehyde‐3‐phosphate dehydrogenase (GAPDH) (Affinity, AF7021, 1:3000), Tubulin (Affinity, AF7011, 1:3000), *β*‐actin (Affinity, AF7018, 1:5000), and then incubated with Goat anti‐Rabbit immunoglobulin G (IgG) (H + L) horseradish peroxidase (HRP) (Affinity, S0001, 1:3000) at room temperature for 1 h. Signal detection was achieved through an enhanced chemiluminescence system, and western blot quantification was performed with Image J.

### 2.10 Immunofluorescence Staining

Bronchial epithelial cell monolayers on coverslips were fixed in 4% paraformaldehyde for 15 min to preserve cellular morphology and antigenicity, then permeabilized in phosphate buffered saline (PBS) (Gibco, US, C10010500BT) containing Triton X‐100 (Solarbio, Beijing, China, T8200) for 10 min at room temperature and washed three times in PBS (5 min each). For single‐label immunofluorescence, coverslips were incubated overnight at 4°C with either anti‐ITGA5 (HuaBio, ET1701‐58; 1:200) or anti‐TrkB (Affinity, AF6461; 1:100). Following three PBS washes, specimens were incubated for 1 h at room temperature with HRP‐conjugated secondary antibody (Pinuofei Biological, Wuhan, China, PN0046), counterstained with 4′,6‐Diamidino‐2′‐phenylindole (DAPI) (Pinuofei Biological, Wuhan, China, PN0015) for 5 min, and mounted. Whole‐slide fluorescence was then acquired using a Pannoramic 250 Flash II Slide Scanner (3DHISTECH, Hungary), and relative fluorescence intensity was quantified in ImageJ. For multi‐label co‐localisation, nonspecific binding was blocked with 10% normal goat serum (Pinuofei Biological, Wuhan, China, PN0038) at 37°C for 30 min. Primary antibodies against ITGA5 (HuaBio, ET1701‐58; 1:200), PI3K (Affinity, AF6242; 1:400), TrkB (Affinity, AF6461; 1:100), and EpCAM (Affinity, DF6311; 1:100) were mixed in antibody diluent and incubated overnight at 4°C. After three PBS washes, HRP‐conjugated goat anti‐rabbit IgG (1:1000 in phosphate buffered saline with tween (PBST) containing 0.003% H_2_O_2_) was applied for 1 h, followed by tyramide signal amplification kits (TYR‐594, TYR‐430, TYR‐555 and TYR‐651, Pinuofei Biological, Wuhan, China; 1:1000) at 37°C for 30 min, washing with PBS between steps. Nuclei were counterstained with DAPI for 5 min, and then coverslips were mounted with antifade medium (Pinuofei Biological, Wuhan, China, PN0024). High‐resolution images were captured on a Leica TCS SPE II confocal microscope under identical exposure settings.

### 2.11 Immunohistochemistry Staining

Paraffin‐embedded tissue sections were deparaffinised and rehydrated, followed by antigen retrieval using ethylenediaminetetraacetic acid buffer under high pressure. Endogenous peroxidase activity was blocked with 3% hydrogen peroxide. Sections were incubated overnight at 4°C with primary antibodies against ITGA5 (Abcam, ab150361, 1:100) or TrkB (Affinity, AF6461, 1:100), followed by incubation with the appropriate secondary antibodies. Immunoreactivity was visualised using 3,3′‐diaminobenzidine, and nuclei were counterstained with haematoxylin. Images were acquired using a Pannoramic 250 Flash II digital slide scanner (3DHISTECH, Hungary), and immunostaining signals were quantified using ImageJ software.

### 2.12 Surface Plasmon Resonance (SPR) Experiment

ITGA5 (TargetMol, TMPJ‐00810) was covalently immobilised on a CM5 sensor chip (Cytiva, BR‐1005‐30) using the standard 1‐ethyl‐3‐(3‐dimethylaminopropyl) carbodiimide/N‐hydroxysuccinimide amine coupling chemistry. The reference channel underwent the same activation and blocking process but without protein injection to correct for non‐specific signals. The running/interaction buffer was 1 × PBS‐P+ (pH 7.4), with 5% (v/v) dimethyl sulfoxide added during the interaction phase. PI3K (IPODIX, PAX2000‐10328) was injected at increasing concentrations in a multi‐gradient setup at a flow rate of 30 μL/min for 60 s per concentration. After each injection, the surface was regenerated with 10 mM glycine hydrochloride (pH 2.0) for 5 min to ensure the surface could be reused and to minimise any residual binding that might affect subsequent measurements. The data were globally fitted to a 1:1 Langmuir binding model after subtracting the reference channel subtraction.

### 2.13 Animals and Treatment

Female BALB/c mice (6–8 weeks old) were purchased from Guangdong Weitong Lihua Laboratory Animal Technology Co. Ltd. (Licence number: SCXK (Yue) 2022‐0063). All animal procedures were reviewed and approved by the Ethics Committee of the Animal Experiment Center at Guangzhou University of Chinese Medicine (Approval number: 20251023007). Mice were randomly divided into the following groups: control, HDM, Res‐L, Res‐H and M200. Following light isoflurane anaesthesia, the asthma mouse model was induced by intranasal instillation of HDM (Greer Laboratories, USA, XPB82D3A25) extract, with 25 μg of HDM administered daily for 5 consecutive days each week over a period of 5 weeks. One week after HDM sensitization, the Res‐L group (50 mg/kg, oral gavage; Selleck, US, S1396), Res‐H group (100 mg/kg, oral gavage; Selleck, US, S1396), and M200 group (10 mg/kg, intraperitoneal injection; TargetMol, T9901A‐008) received drug interventions 1 h prior to allergen challenge.

### 2.14 Pathological Staining

Paraffin‐embedded tissue sections were deparaffinised in xylene, followed by dehydration through a graded ethanol series, and cut into 5 μm‐thick sections. Haematoxylin and eosin (H&E) staining was performed to assess the extent of inflammatory cell infiltration in the lung and peribronchial tissues. Periodic acid‐schiff (PAS) staining was used to evaluate mucus secretion in the airways. Collagen deposition in the lung and peribronchial areas was visualised by Masson's trichrome staining. Whole‐slide images were acquired using a Pannoramic 250 Flash II Slide Scanner (3DHISTECH, Hungary). The pathology score for each sample was calculated as the average of three random field scores.

### 2.15 Measurement of Airway Resistance and Lung Compliance

24 h after the final HDM challenge, airway resistance and lung compliance were assessed using the Buxco pulmonary function system. Airway hyperresponsiveness was induced with methacholine. Specifically, inhalation of 0 mg/mL methacholine was used as the baseline control to eliminate any interference from the nebulization process on airway function and to provide a reference for subsequent dose‐dependent responses. The concentration of nebulized methacholine was then escalated sequentially (3.125 mg/mL, 6.25 mg/mL, 12.5 mg/mL and 25 mg/mL), and airway resistance and dynamic lung compliance were measured using direct tracing after each dose.

### 2.16 Bronchoalveolar Lavage Fluid (BALF) Collection and Enzyme‐Linked Immunosorbent Assay (ELISA)

BALF was collected and processed as previously described [44]. The pellets were suspended for total cell counting using a Countstar (BioLab) counter. BALF was used to detect total IgE and inflammatory cytokine levels. The secretion levels of IgE (Neobioscience, EMC117), IL‐4 (Neobioscience, EMC003), IL‐5 (Neobioscience, EMC108) and IL‐13 (Neobioscience, EMC124) were determined using ELISA assays according to the manufacturer's protocols.

### 2.17 Statistical Analysis

Statistical analyses were conducted in GraphPad Prism 10, with results expressed as mean ± standard error of the mean (SEM). Each assay was performed three times. Normality was assessed using the Shapiro–Wilk test. For comparisons between two groups, an unpaired two‐tailed Student's *t*‐test was used for normally distributed data, whereas the Mann–Whitney U test was applied for non‐normally distributed data. For comparisons among multiple groups, one‐way ANOVA was performed for normally distributed data with homogeneity of variances; Welch's ANOVA was used when variances were unequal. For non‐normally distributed data, the Kruskal‐Wallis test was applied. Correlation analyses were performed using Spearman's rank correlation coefficient. Statistical significance was defined as *p* < 0.05.

## 3 Results

### 3.1 ITGA5 Was Upregulated in Airway Epithelial Cells of Asthma Patients and TGF‐*β*1‐Induced EMT Models

**FIGURE 1:**
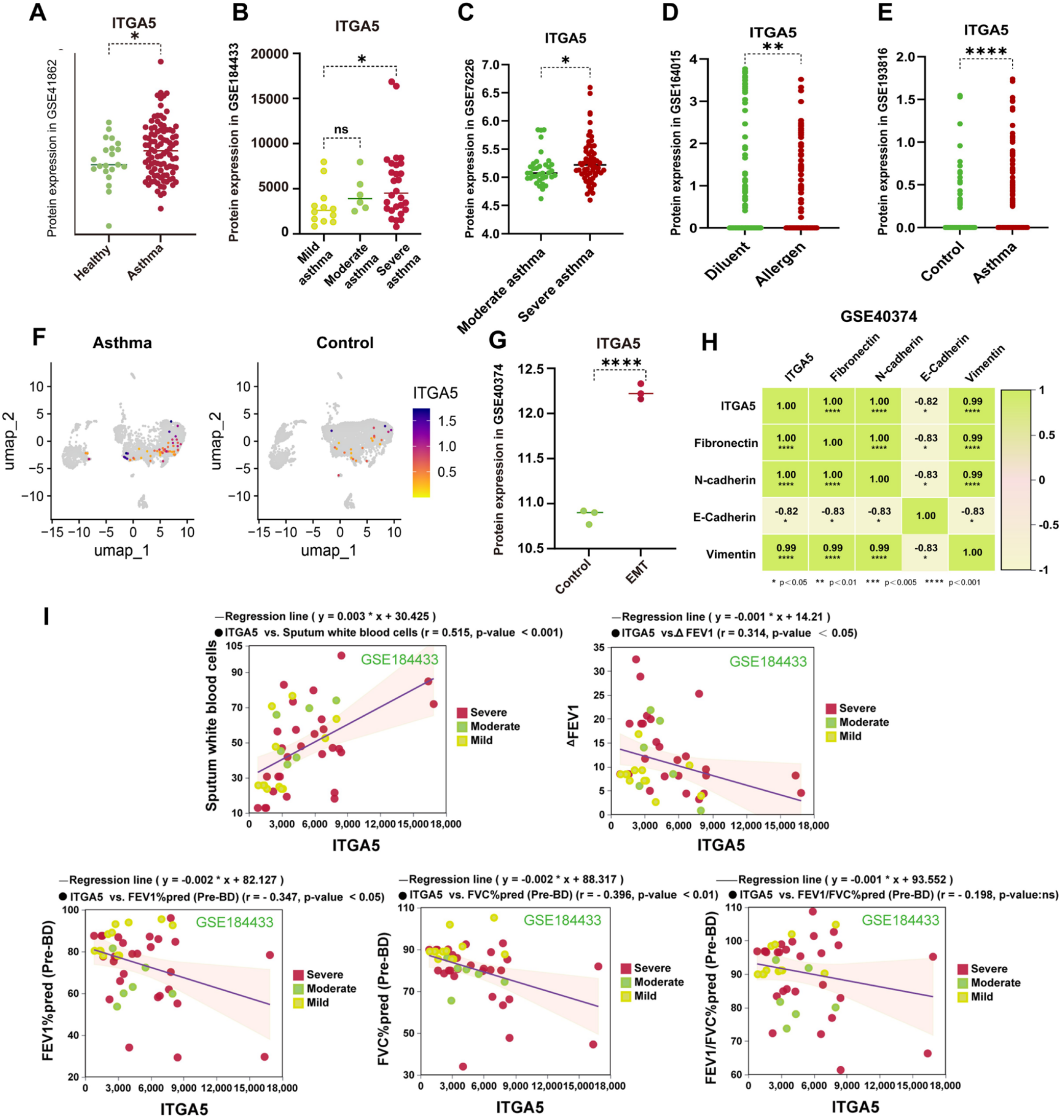
Upregulated ITGA5 expression in the airways of asthma patients and in a TGF‐*β*1‐induced EMT model, and its association with impaired lung function. (A) ITGA5 expression in bronchial brushings from asthma patients compared with healthy volunteers (GSE41862). (B) ITGA5 expression in sputum airway cells from patients with mild, moderate, and severe asthma (GSE184433). (C) ITGA5 expression in bronchial brushing samples from moderate and severe asthma patients (GSE76226). (D) ITGA5 expression in bronchial brushing samples from asthma patients (GSE164015). (E) ITGA5 expression in airway mucosal samples from asthma patients and allergic non‐asthma controls (GSE193816). (F) Differential distribution of ITGA5 in airway mucosal samples from asthma patients and allergic non‐asthma controls shown in a umap plot (GSE193816). (G) ITGA5 expression in an in vitro TGF‐*β*1‐induced EMT model using human bronchial epithelial cells (GSE40374). (H) Correlation heatmap between ITGA5 and mesenchymal markers (Fibronectin, N‐cadherin, Vimentin) and the epithelial marker E‐cadherin (GSE40374). (I) Scatter plots depicting correlations between ITGA5 expression and sputum total leukocyte count, and lung function parameters: ΔFEV_1_, FEV_1_ % predicted, FVC % predicted, and FEV_1_/FVC % predicted in asthma patients (GSE184433). Data were presented as mean ± SEM. *p < 0.05, **p < 0.01, ***p < 0.001, ****p < 0.0001.

Gene expression profiling of airway scrapings obtained from asthma patients compared with healthy controls revealed significantly elevated ITGA5 expression in asthma. Transcriptomic analysis of sputum cells and bronchial brush samples further demonstrated that ITGA5 expression was higher in patients with severe asthma than in those with mild or moderate asthma, suggesting an association between ITGA5 expression and disease severity (Figure 1A–C). Single‐cell analysis further confirmed the high expression of ITGA5 in the airways of asthma patients, and the expression pattern showed a distinct dimensional reduction distribution compared to allergic non‐asthma controls (Figure 1D–F). In a TGF‐*β*1‐induced bronchial EMT model, ITGA5 expression was upregulated and favorably correlated with mesenchymal markers (Fibronectin, N‐cadherin and Vimentin), but negatively correlated with E‐cadherin (Figure 1G,H). These findings support that ITGA5 promotes airway remodelling, a hypothesis that was validated in vitro and in vivo. Correlation analysis revealed that ITGA5 expression was positively associated with total sputum leukocytes (Figure 1I). Clinically, higher ITGA5 levels were strongly correlated with impaired lung function, as indicated by changes in forced expiratory volume in 1 s (ΔFEV₁), forced expiratory volume in 1 s (FEV_1_) % predicted, forced vital capacity (FVC) % predicted, and FEV_1_/FVC % predicted (Figure 1I).

### 3.2 TGF‐*β*1 Induced EMT Phenotype in Bronchial Epithelial Cells

**FIGURE 2:**
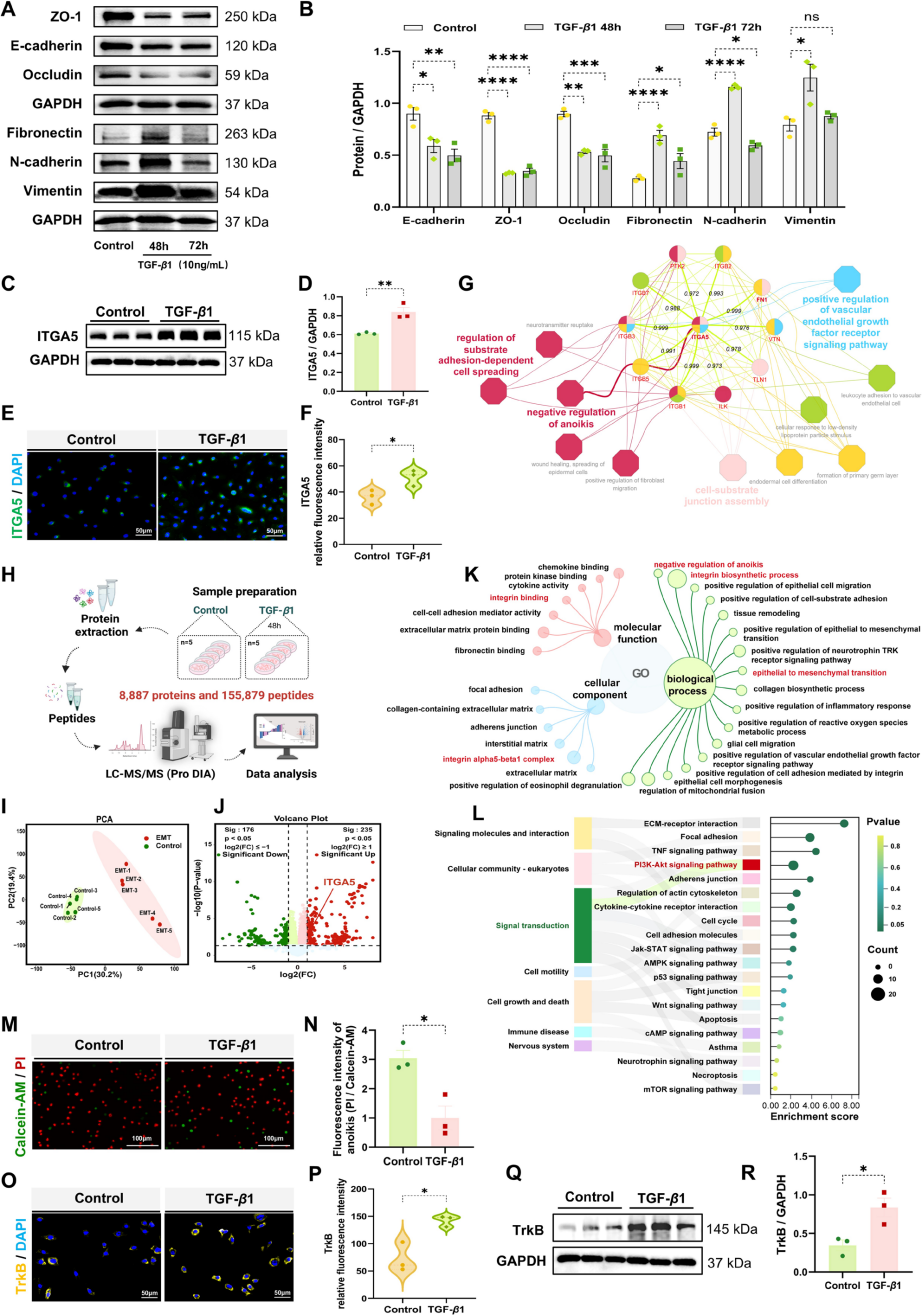
TGF‐*β*1 induced EMT and anoikis resistance in human bronchial epithelial cells (HBE135‐E6E7). (Aand B) Western blot analysis of EMT phenotype markers (ZO‐1, E‐cadherin, Occludin, Fibronectin, N‐cadherin, Vimentin) at 48 h and 72 h, respectively. (C and D) Protein expression level of ITGA5 in the TGF‐*β*1‐induced HBE135‐E6E7 cell EMT model. (E and F) Fluorescence expression intensity of ITGA5 in the TGF*‐β*1‐induced HBE135‐E6E7 cell EMT model scale bar = 50 μm. (G) Protein interaction network of ITGA5 predicted by STRING. (H) Pro DIA‐based quantitative proteomics analysis process (*n* = 5). (I) PCA analysis of all samples. (J) Volcano plot, red and green dots denote significantly up‐ and down‐regulated differentially expressed proteins (DEPs). DEPs identification criteria: | log_2_FC | ≥ 1 and *p* < 0.05. (K) GO enrichment analysis of DEPs, enrichment threshold: *P* < 0.05. (L) KEGG enrichment analysis of DEPs, enrichment threshold: *P* < 0.05. (M and N) Anoikis assay: Green calcein‐AM fluorescence (Ex = 485 nm, Em = 516 nm) indicates viable cells independent of adhesion, while red PI fluorescence (Ex = 535 nm, Em = 617 nm) indicates anoikis‐induced cell death. Scale bar: 100 μm. (O and P) Fluorescent intensity of the anoikis resistance marker TrkB. Scale bar: 50 μm. (Q and R) Protein levels of the anoikis resistance marker TrkB. Data were presented as mean ± SEM. *n* = 3 for each group. *p < 0.05, **p < 0.01, ***p < 0.001, ****p < 0.0001.

TGF*‐β*1 is a key driver of EMT and airway remodelling in asthma. Following established protocols, human bronchial epithelial cells were stimulated with 10 ng/mL TGF‐*β*1, and EMT phenotypic markers were assessed at different time points (48 h, 72 h). In line with earlier research, TGF‐*β*1 stimulation for 48 h significantly downregulated the epithelial indicators E‐cadherin, tight junction proteins ZO‐1 and Occludin, while significantly upregulating the mesenchymal markers Fibronectin, N‐cadherin and Vimentin (Figure 2A,B). Since these changes were more pronounced at 48 h than at 72 h, 48 h was chosen as the ideal time point for further research. 48 h after TGF‐*β*1 exposure, EMT cells had considerably higher levels of ITGA5 than untreated controls (Figure 2C–F). Interestingly, ITGA5 is functionally clustered in biological pathways such as negative regulation of anoikis, wound healing, spreading of epidermal cells, and positive regulation of vascular endothelial growth factor receptor signalling pathway. Computational prediction of ITGA5 protein interactors further identified strong associations with EMT‐regulatory proteins, including Fibronectin and members of the integrin family (Figure 2G).

### 3.3 Proteomic Profiling Identified DEPs Enriched in the Negative Regulation of Anoikis

LC–MS/MS analysis coupled with database searching identified a total of 8887 proteins and 155,879 peptides across 10 samples (Figure 2H). PCA analysis revealed high similarity within groups and evident distinction between groups, demonstrating different protein expression variations in bronchial epithelial cells induced by TGF‐*β*1 (Figure 2I). Differential analysis identified 235 proteins, including ITGA5, that were significantly upregulated in the EMT model, and 176 proteins that were significantly downregulated (Figure 2J). We focused on the biological functions and signalling pathways associated with the upregulated DEPs. GO enrichment analysis revealed that the elevated DEPs were significantly enriched in cellular components such as the integrin *α*5*β*1 complex, adherens junctions, and the extracellular matrix. These proteins primarily exhibited molecular functions including integrin binding and extracellular matrix protein binding, and were engaged in biological processes such as epithelial to mesenchymal transition, negative regulation of anoikis, and integrin biosynthetic processes (Figure 2K). KEGG pathway analysis indicated that the upregulated DEPs were notably abundant in the PI3K/Akt signalling pathway, which promotes cell proliferation, growth and survival, while inhibiting apoptosis (Figure 2L). Interestingly, the TGF‐*β*1‐induced EMT model appears to be closely linked to integrin‐mediated signalling and the negative control of anoikis. Anoikis assays proved a significant decrease in the mean fluorescence intensity ratio of Calcein‐AM/PI in the TGF‐*β*1‐treated group compared to the control group, indicating that TGF‐*β*1‐induced bronchial epithelial cells exhibited anoikis resistance (Figure 2M,N). Furthermore, consistent changes were observed in the expression of anoikis resistance markers, with a notable upregulation of TrkB protein levels and fluorescence intensity in the TGF‐*β*1‐induced EMT model compared to the control (Figure 2O–R). The accumulation of TrkB indicated the existence of anoikis resistance following TGF‐*β*1 induction.

### 3.4 ITGA5 Might Serve as a Potential Predictive Marker for Anoikis Resistance in the TGF‐*β*1‐Induced EMT Model

**FIGURE 3:**
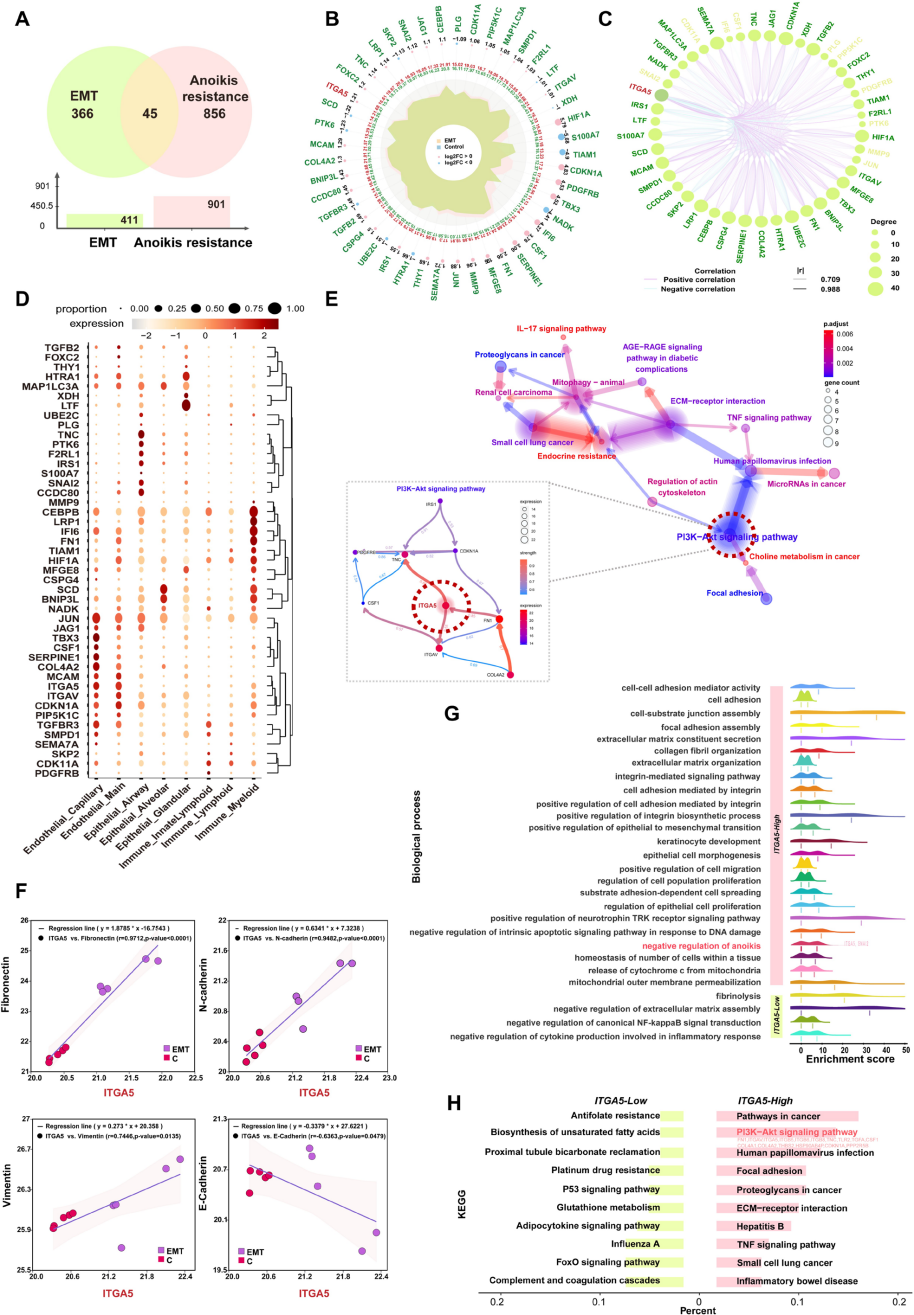
ITGA5 was associated with both TGF‐*β*1‐induced EMT and anoikis resistance in bronchial epithelial cells. (A) Venn plot of DEPs involved in EMT and anoikis resistance gene set identified 45 anoikis resistance‐related genes (ARRGs). (B) Radar plot of protein expression levels of ARRGs. (C) Co‐expression network of ARRGs. The size of the node represents the degree value. The larger the degree, the more genes interact with it. The connecting lines represent the correlation between ARRGs. The red line represents positive correlation and the blue line represents negative correlation. (D) Bubble plot of cell expression of ARRGs in healthy human lungs. (E) Pathway regulatory network of ARRGs. (F) Scatter plots of the correlation between ITGA5 and mesenchymal markers Fibronectin, N‐cadherin, Vimentin and epithelial marker E‐cadherin. (G) Biological process enrichment analysis of ITGA5‐high and ITGA5‐low groups, enrichment threshold: *P* < 0.05. (H) KEGG enrichment analysis of ITGA5‐high and ITGA5‐low groups, enrichment threshold: *P* < 0.05.

After retrieving a gene set associated with anoikis resistance and intersecting it with the DEPs, we found a total of 45 overlapping targets (Figure 3A). Among these, 33 anoikis resistance‐related genes (ARRGs) were upregulated, while 12 were downregulated in the EMT model (Figure 3B). Co‐expression network analysis revealed strong interconnections among these 45 ARRGs. Further exploration of the relationship between ITGA5 and ARRGs indicated significant positive correlations with most ARRGs (Figure 3C). Notably, all 45 ARRGs were annotated in the airway epithelium of normal human lungs (Figure 3D). To further investigate the potential regulatory mechanisms of ARRGs, we used the CBNplot algorithm for Bayesian network inference of signalling pathways. ITGA5 was enriched within the PI3K/Akt signalling pathway, and Fibronectin was identified as a potential upstream regulator of ITGA5 (Figure 3E). Consistent with these findings, our proteomic data demonstrated a significant positive correlation between ITGA5 and EMT markers (Fibronectin, N‐cadherin, Vimentin), as well as a significant negative correlation with E‐cadherin (Figure 3F). Subsequent differential analysis based on median ITGA5 expression levels showed that the ITGA5‐high group was substantially enriched in biological processes such as integrin‐mediated cell adhesion, positive regulation of epithelial‐mesenchymal transition, negative regulation of anoikis, and positive regulation of neurotrophin TRK receptor signalling pathway, as well as pathways including PI3K/Akt signalling, focal adhesion, and ECM‐receptor interaction (Figure 3G,H). All of these findings point to a crucial role for ITGA5 in EMT, possibly via modulation of the PI3K/Akt pathway and negative control of anoikis.

### 3.5 Interactions and Co‐Localised Expression Among ITGA5, PI3K and TrkB

**FIGURE 4:**
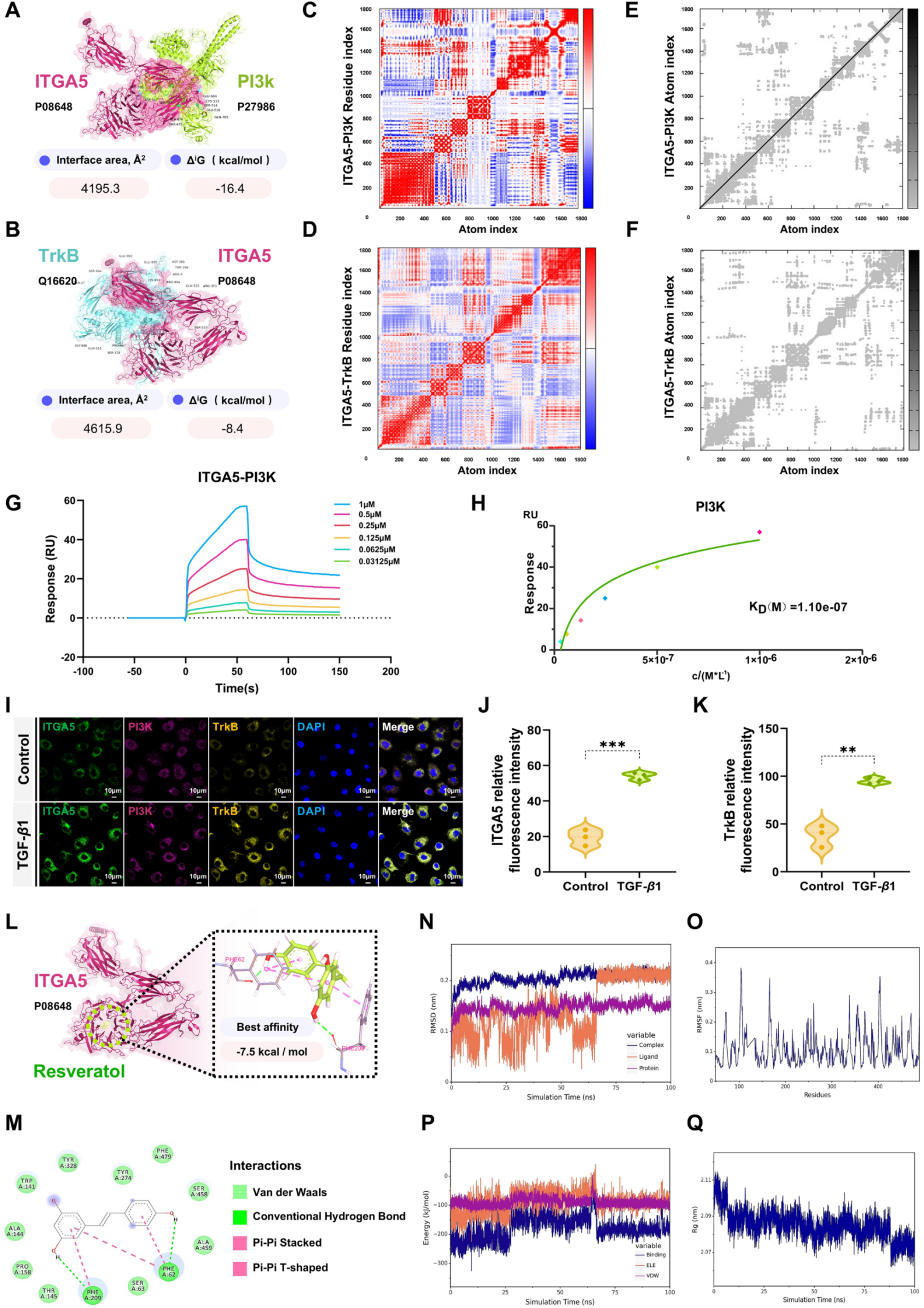
Protein interaction analyses of ITGA5 with PI3K and TRKB and MD simulation of the Res‐ITGA5 complex. (A and B) Representative docked conformations of ITGA5 with PI3K and ITGA5 with TrkB, respectively. (C and D) Covariance matrices derived from NMA: Red regions denote positively correlated motions between residue pairs, whereas blue regions denote negatively correlated (anti‐correlated) motions. (E and F) Elastic network models illustrating the rigidity of interfacial atoms (grey regions), which resist deformation during collective motions. (G and H) SPR analysis of the protein–protein interaction between ITGA5 and PI3K. (I and K) Cellular expression localisation and fluorescence intensity of ITGA5 (green), PI3K (magenta), and TrkB (yellow). Scale bar: 10 μm. (L and M) Representative docking conformations of the Res‐ITGA5 complex, together with interaction mapping and hydrogen bond analysis. (N) RMSD plot of the Res‐ITGA5 complex. (O) RMSF analysis of the Res‐ITGA5 complex. (P) Binding energy profile of the Res‐ITGA5 complex, in which VDW represents van der Waals and hydrophobic interactions, ELE denotes electrostatic interactions, and Binding corresponds to the sum of VDW and ELE without considering solvation effects. (Q) Rg plot curve characteristics. Data were presented as mean ± SEM. *n* = 3 for each group. *p < 0.05, **p < 0.01, ***p < 0.001, ****p < 0.0001.

Building on the previously described findings, we further performed an exploratory analysis of potential interactions between ITGA5 and PI3K, and between ITGA5 and the anoikis‐resistance marker TrkB. PDBePISA analysis of the top‐ranked protein–protein complex models predicted by GRAMM revealed extensive interface areas and highly stable interaction energies for both ITGA5‐PI3K and ITGA5‐TrkB complexes (Figure 4A,B). Subsequent NMA further confirmed the favourable dynamic coupling between ITGA5 and each partner. The covariance matrix highlighted broad regions of significantly correlated residue motions (red zones), whereas the elastic network model demonstrated strong rigidity along the interaction trajectories (grey zones), collectively supporting the complexes' resistance to conformational deformation during their dynamic fluctuations (Figure 4C–F). SPR experiments, which demonstrated a stable and well‐characterised direct interaction between ITGA5 and PI3K (*K*_*D*_ = 7.43e‐07 M), provided more proof of the two proteins' capacity for direct binding (Figure 4G,H). Subsequent immunofluorescence analysis demonstrated that TGF‐*β*1 induction markedly increased the fluorescence intensity of ITGA5 and TrkB in bronchial epithelial cells compared to the control. Furthermore, robust co‐localisation signals between ITGA5, PI3K and TrkB (Figure 4I–K) provided direct visual evidence of their interaction, suggesting that ITGA5 may control anoikis resistance by modulating PI3K signalling.

### 3.6 Resveratrol and M200 Were Identified as Candidate Compounds for Targeted Intervention of ITGA5

However, there are no direct macromolecular biopharmaceuticals or small‐molecule drugs targeting ITGA5 currently available. As a humanised monoclonal antibody, M200 has the pharmacological ability to inhibit ITGA5 by specifically blocking the binding of *α*5*β*1 to Fibronectin. Notably, CTD drug prediction revealed that Res, as the top‐ranked natural small‐molecule plant compound, might possess potential pharmacological inhibitory potential (Figure S1A). The binding affinity of 10 dockings was > −6 kcal/mol (Figure S1B), and the best affinity was −7.5 kcal/mol (Figure 4L). Molecular docking results showed that the amino acids PHE‐62 and PHE‐209 of ITGA5 formed hydrogen bonds, Pi‐Pi Stacked and Pi‐Pi T‐shaped hydrophobic interactions with Res, while amino acids such as SER‐63, ALA‐144, and PHE 479 of ITGA5 formed van der Waals interactions with the small molecule (Figure 4M). Simulation results indicated that the root mean square deviation (RMSD) of the docking complex structure gradually stabilised with the progress of the simulation; the root mean square fluctuation (RMSF) was small, and the flexibility of surrounding amino acids was low; the van der Waals forces (VDW) and electrostatic interactions (ELE) in the complex gradually stabilised with the simulation; and the radius of gyration (Rg) curve fluctuated slightly within a certain range and reached dynamic equilibrium as the simulation progressed (Figure 4N–Q). Analysis of the binding energy and its components of the complex in the equilibrium state confirmed the strong binding energy and affinity between the two molecules, which further supported the potential pharmacological inhibitory capacity of Res against ITGA5.

### 3.7 Sh‐ITGA5 Inhibited the TGF‐*β*1‐Induced EMT Phenotype, Attenuated Activation of the PI3K/Akt Signalling Pathway and Alleviated Anoikis Resistance in Bronchial Epithelial Cells

**FIGURE 5:**
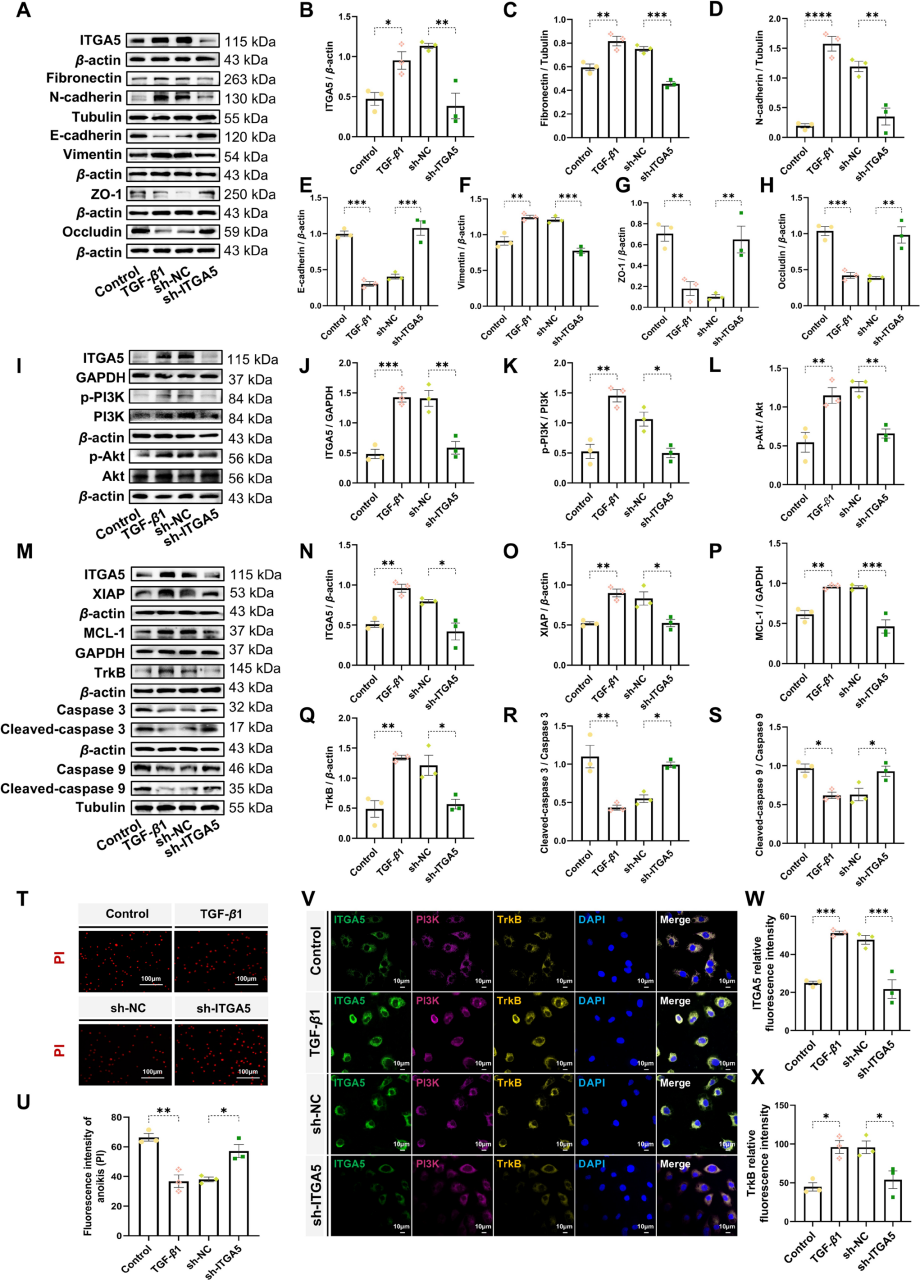
Effects of ITGA5 knockdown on TGF‐*β*1‐induced EMT, PI3K/Akt signalling, and anoikis resistance in bronchial epithelial cells. (A–H) Western blot analysis of protein expression levels of EMT markers (Fibronectin, N‐cadherin, E‐cadherin, Vimentin, ZO‐1 and Occludin) in TGF‐*β*1 stimulated cells following ITGA5 knockdown. (I–L) Western blot analysis of PI3K/Akt signalling axis proteins following ITGA5 knockdown. (M–S) Western blot analysis of anoikis resistance related proteins (XIAP, MCL‐1, TrkB, Caspase 3, Cleaved‐caspase 3, Caspase 9 and Cleaved‐caspase 9) after ITGA5 knockdown. (T–U) Anoikis assay post‐ITGA5 knockdown: Red PI fluorescence (Ex = 535 nm, Em = 617 nm) indicates anoikis‐induced cell death; scale bar: 100 μm. (V–X) Cellular spatial colocalisation and fluorescence intensity analyses of ITGA5 (green) with PI3K (magenta) and TrkB (yellow) after ITGA5 knockdown (*n* = 3); scale bar: 10μm. Data were presented as mean ± SEM. *n* = 3 for each group. *p < 0.05, **p < 0.01, ***p < 0.001, ****p < 0.0001.

To further elucidate the pathogenic role of ITGA5 in asthma‐associated EMT, we conducted targeted loss‐of‐function studies. HBE135‐E6E7 cells were transduced using three lentiviral shRNA constructs targeting ITGA5 and one control vector (Figure S2A). Given its superior transduction efficiency and potent suppression of ITGA5 protein, sh‐ITGA5#2 was chosen to establish a stable ITGA5‐knockdown cell line (Figure S2B–D). Western blot results revealed that sh‐ITGA5 substantially suppressed the EMT phenotype triggered by TGF‐*β*1 in bronchial epithelial cells. Specifically, compared to the TGF‐*β*1‐treated vector control (sh‐NC), downregulation of ITGA5 resulted in decreased expression of EMT‐promoting proteins, such as Fibronectin (ECM regulator), N‐cadherin (mesenchymal cell–cell adhesion), and Vimentin (cytoskeletal component). Meanwhile, epithelial integrity markers, including E‐cadherin, ZO‐1 and Occludin, were significantly upregulated (Figure 5A–H). These coordinated changes facilitated the reversion of bronchial epithelial cells from an EMT phenotype towards a normal epithelial state. The activation of the PI3K/Akt signalling cascade greatly amplified the TGF‐*β*1‐induced EMT impact in bronchial epithelial cells. Phosphorylation of PI3K and Akt is essential for their full kinase activity, and increased p‐PI3K and p‐Akt levels directly reflect activation of the signalling axis. TGF‐*β*1 treatment markedly elevated p‐PI3K and p‐Akt levels in our experiments relative to untreated controls, suggesting that the PI3K/Akt pathway was activated in bronchial epithelial cells. sh‐ITGA5 significantly attenuated TGF‐*β*1‐driven phosphorylation levels of PI3K and Akt (Figure 5I–L). Simultaneously, we observed that TrkB was significantly increased under TGF‐*β*1 stimulation, accompanied by a significant increase in anti‐apoptotic proteins XIAP and MCL‐1, and a significant decrease in both initiators (Caspase‐9 and Cleaved Caspase‐9) and executioners (Caspase‐3 and Cleaved Caspase‐3) apoptotic proteases (Figure 5M–S). Notably, these changes in proteins collectively indicated a strong tendency of apoptosis inhibition, while ITGA5 knockdown reversed the TGF‐*β*1‐induced apoptosis resistance phenotype. Moreover, anoikis assays confirmed that sh‐ITGA5 restored apoptotic activity in bronchial epithelial cells, thereby reversing the TGF‐*β*1‐induced resistance to anoikis (Figure 5T,U). Consistent with our other findings, immunofluorescence analysis demonstrated that, under TGF‐*β*1 stimulation, ITGA5 exhibited pronounced intracellular spatial colocalisation with PI3K and TrkB, indicating potential similarities in their subcellular distribution patterns. This co‐localisation effect was markedly attenuated by sh‐ITGA5 intervention (Figure 5V–X). These findings indicated that in the TGF‐*β*1‐driven EMT model, ITGA5 knockdown might have deactivated the PI3K/Akt pathway, reducing anoikis resistance in bronchial epithelial cells.

### 3.8 Res and M200 Pharmacologically Inhibited ITGA5, Improved EMT and Reduced PI3K/Akt Signalling Pathway Activation and Anoikis Resistance in Bronchial Epithelium Cells

**FIGURE 6:**
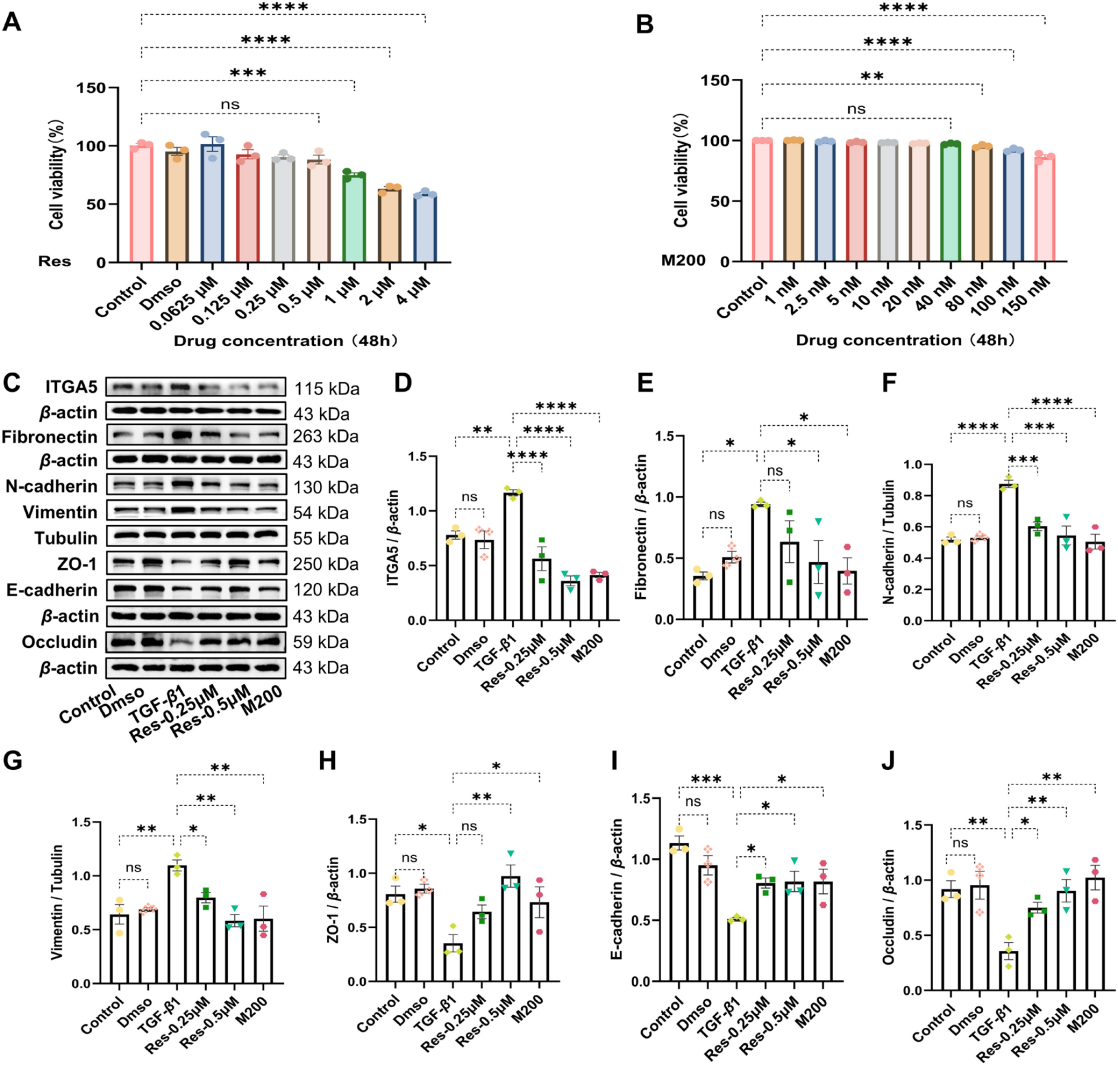
Res and M200 pharmacologically inhibited ITGA5 and ameliorated TGF‐*β*1–induced EMT in bronchial epithelium cells. (A and B) Cell viability of HBE135‐E6E7 cells following 48 h treatment with varying concentrations of Res or M200, as measured by CCK‐8 assay. (C–J) Res and M200 inhibited ITGA5 and improved the expression of EMT markers (Fibronectin, N‐cadherin, Vimentin, ZO‐1, E‐cadherin, and Occludin) proteins. Data were presented as mean ± SEM. *n* = 3 for each group. *p < 0.05, **p < 0.01, ***p < 0.001, ****p < 0.0001.

**FIGURE 7:**
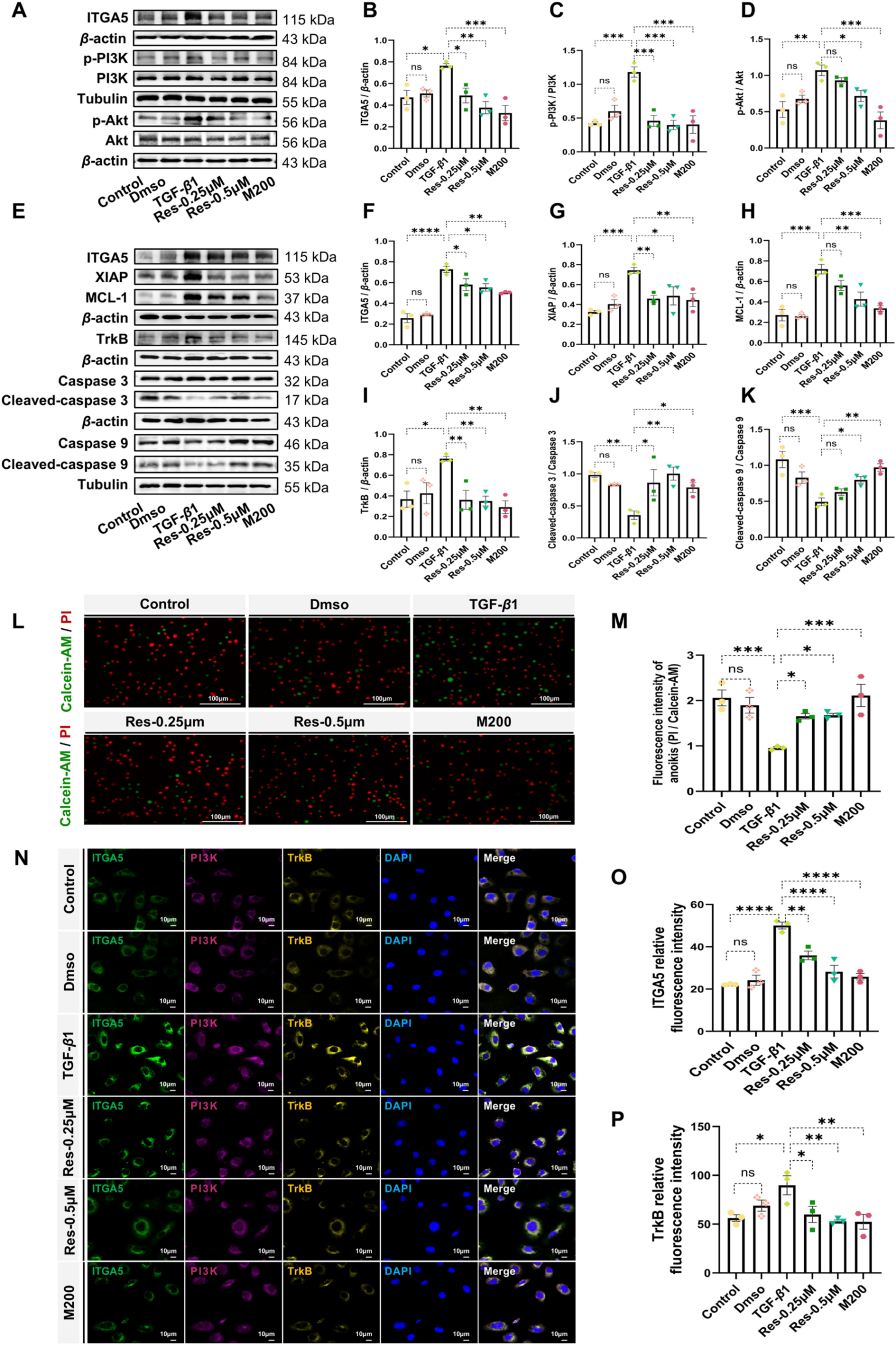
Res and M200 inhibited ITGA5, suppressed PI3K/Akt pathway activation, and alleviated anoikis resistance. (A–D) Western blot analysis of PI3K/Akt signalling axis proteins in cells treated with Res or M200. (E–K) Western blot analysis of anoikis resistance related proteins (XIAP, MCL‐1, TrkB, Caspase 3, Cleaved‐caspase 3, Caspase 9 and Cleaved‐caspase 9) in cells treated with Res or M200. (L and M) Effects of Res and M200 on anoikis: Green calcein‐AM fluorescence (Ex = 485 nm, Em = 516 nm) indicates viable cells independent of adhesion, red PI fluorescence (Ex = 535 nm, Em = 617 nm) marked anoikis‐induced cell death, scale bar: 100 μm. (N–P) Spatial colocalisation and fluorescence intensity analyses of ITGA5 (green) with PI3K (magenta) and TrkB (yellow); scale bar: 10 μm. Data were presented as mean ± SEM. *n* = 3 for each group. *p < 0.05, **p < 0.01, ***p < 0.001, ****p < 0.0001.

As potential pharmacological inhibitors of ITGA5, Res and M200 played critical roles in suppressing TGF‐*β*1‐induced EMT. This study first assessed the cytotoxicity of Res and M200 on normal bronchial epithelial cells using a CCK‐8 assay. Non‐toxic concentrations of Res (0.25 and 0.5 μM) and M200 (40 nM) were then selected to evaluate their pharmacological inhibition of ITGA5 and consequent effects on EMT (Figure 6A,B). Interestingly, both Res and M200 markedly reduced ITGA5 protein levels and, in parallel, suppressed the mesenchymal markers Fibronectin, N‐cadherin and Vimentin. Conversely, expression of the epithelial integrity markers ZO‐1, E‐cadherin and Occludin was significantly upregulated (Figure 6C–J). These coordinated changes effectively blocked the EMT process in bronchial epithelial cells, highlighting Res and M200 as promising therapeutic candidates for targeting ITGA5 to ameliorate asthma‐associated EMT. Res and M200 also prevented TGF‐*β*1 from activating the PI3K/Akt signalling cascade. We found that Res and M200 significantly reduced the kinase activities of PI3K and Akt by considerably lowering their phosphorylation levels while also decreasing ITGA5 protein expression (Figure 7A–D). Meanwhile, Res and M200 downregulated the expression of anti‐apoptotic proteins (XIAP, MCL‐1) and TrkB, while promoting the upregulation of intrinsic apoptosis‐related initiator and effector proteins (Caspase 9, Cleaved‐caspase 9, Caspase 3 and Cleaved‐caspase 3), which effectively reversed the TGF‐*β*1‐induced anoikis resistance phenotype (Figure 7E–K). Furthermore, both agents reduced survival of bronchial epithelial cells under anoikis conditions, favouring anoikis induction and inhibiting anchorage‐independent proliferation (Figure 7L,M). Consistently, different doses of Res and M200 markedly reduced the fluorescence intensity of ITGA5 and TrkB, while also markedly attenuating the intracellular spatial localisation signals of ITGA5, PI3K and TrkB, disrupting their functional interactions (Figure 7N–P). These findings underscore the therapeutic potential of Res and M200 for targeting ITGA5 to reverse EMT and anoikis resistance in asthma.

### 3.9 Res and M200 Inhibited ITGA5 Expression, PI3K/Akt Pathway Activation, and Anoikis Resistance to Alleviated EMT in Asthma Mice

**FIGURE 8:**
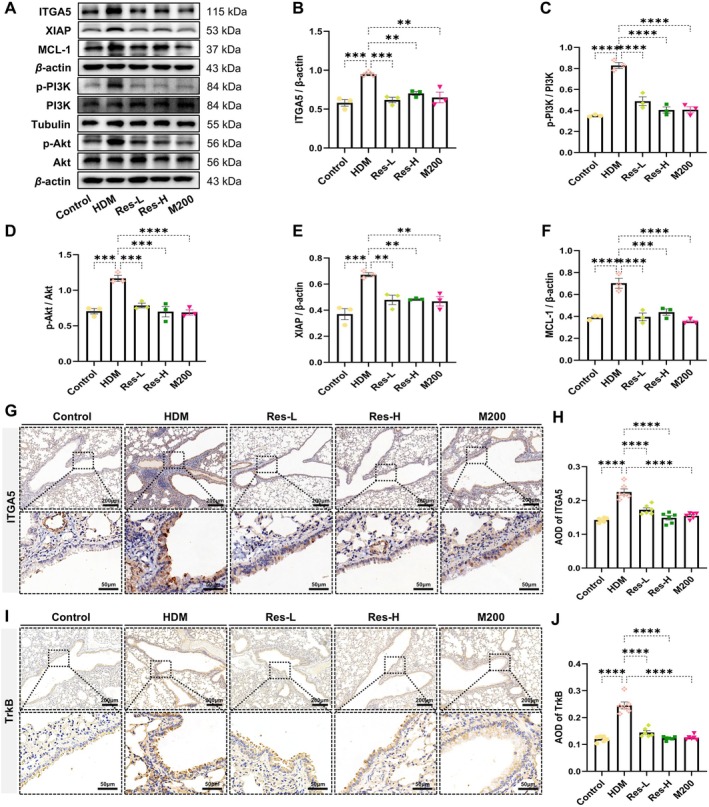
Elevated ITGA5 expression in asthma mice lungs accompanied by upregulation of the PI3K/Akt pathway and anoikis resistance marker TrkB. (A–F) Western blot analysis of ITGA5, PI3K/Akt signalling–related proteins and anoikis resistance–associated proteins (XIAP and MCL‐1) in lung tissues of asthma mice. *n* = 3 for each group. (G–H) Immunohistochemical analysis of ITGA5 localisation and expression in lung tissues of asthma mice. *n* = 6 for each group. Scale bars: 200 and 50 μm. (I–J) Immunohistochemical analysis of TrkB localisation and expression in lung tissues of asthma mice. *n* = 6 for each group. Scale bars: 200 and 50 μm. Data are presented as mean ± SEM. *p < 0.05, **p < 0.01, ***p < 0.001, ****p < 0.0001.

**FIGURE 9:**
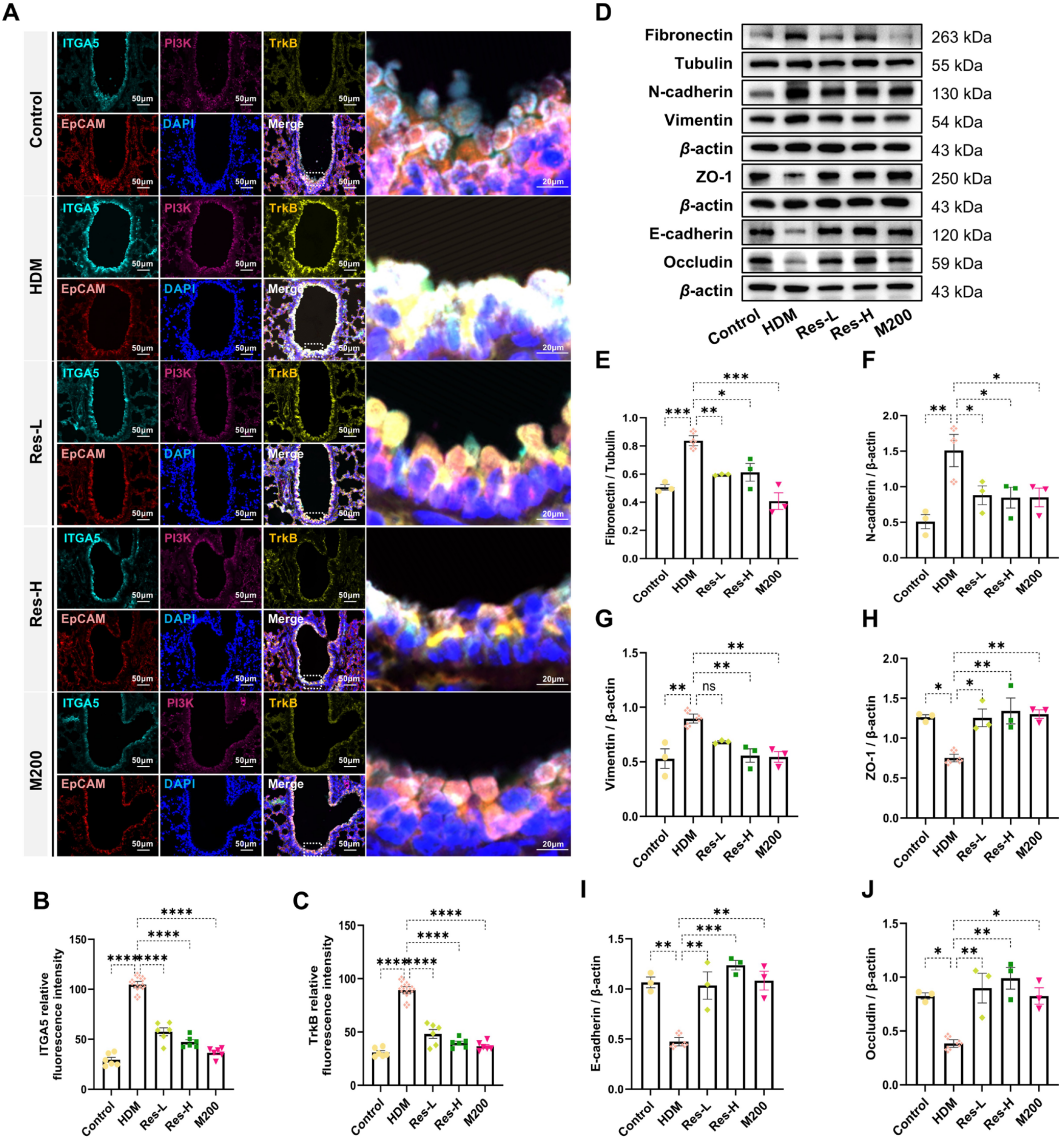
Res and M200 inhibited ITGA5 in vivo and reversed EMT in the lungs of asthma mice. (A–C) Immunofluorescence analysis of the colocalisation and expression of ITGA5, PI3K, and TrkB with the airway epithelial marker EpCAM in the lungs of asthma mice after treatment with Res and M200. *n* = 6 for each group. Scale bars: 50 and 20 μm. (D–J) Western blot analysis of the protein levels of EMT‐related markers (Fibronectin, N‐cadherin, Vimentin, ZO‐1, E‐cadherin, and Occludin) in lung tissues from asthma mice. *n* = 3 for each group. Data are presented as mean ± SEM. *p < 0.05, **p < 0.01, ***p < 0.001, ****p < 0.0001.

An HDM‐sensitised mouse model of asthma was created to further clarify the pathogenic function of ITGA5 in asthma and the pharmacological inhibitory effects of Res and M200. The lungs of asthma mice had higher levels of ITGA5 expression, which was linked to the activation of the PI3K/Akt signalling pathway and the overexpression of anoikis resistance markers (XIAP and MCL1). However, these tendencies were dramatically inhibited by different dosages of Res and M200 (Figure 8A–F). Immunohistochemistry confirmed pronounced positive expression of ITGA5 in the airway epithelium of asthma mice; this increased expression correlated with that of the anoikis resistance marker TrkB. Res and M200 successfully inhibited the elevation of both ITGA5 and TrkB (Figure 8G–J). Immunofluorescence analyses concurrently revealed co‐localisation of ITGA5, PI3K and TrkB with the airway epithelial marker EpCAM in lung tissues of asthma mice, indicating evident anoikis resistance in the airway epithelium. Res and M200 exhibited inhibitory effects consistent with in vitro observations (Figure 9A–C). Res and M200 also suppressed the expression of mesenchymal transition‐promoting proteins (Fibronectin, N‐cadherin and vimentin) in asthma mouse lungs, while reversing downregulation of airway epithelial barrier and adherens junction markers (ZO‐1, E‐cadherin and Occludin), ultimately mitigating EMT in the lungs (Figure 9D–J).

### 3.10 Res and M200 Alleviated Airway Pathological Structural Changes and Improved Lung Function in HDM‐Sensitised Asthma Mice

**FIGURE 10:**
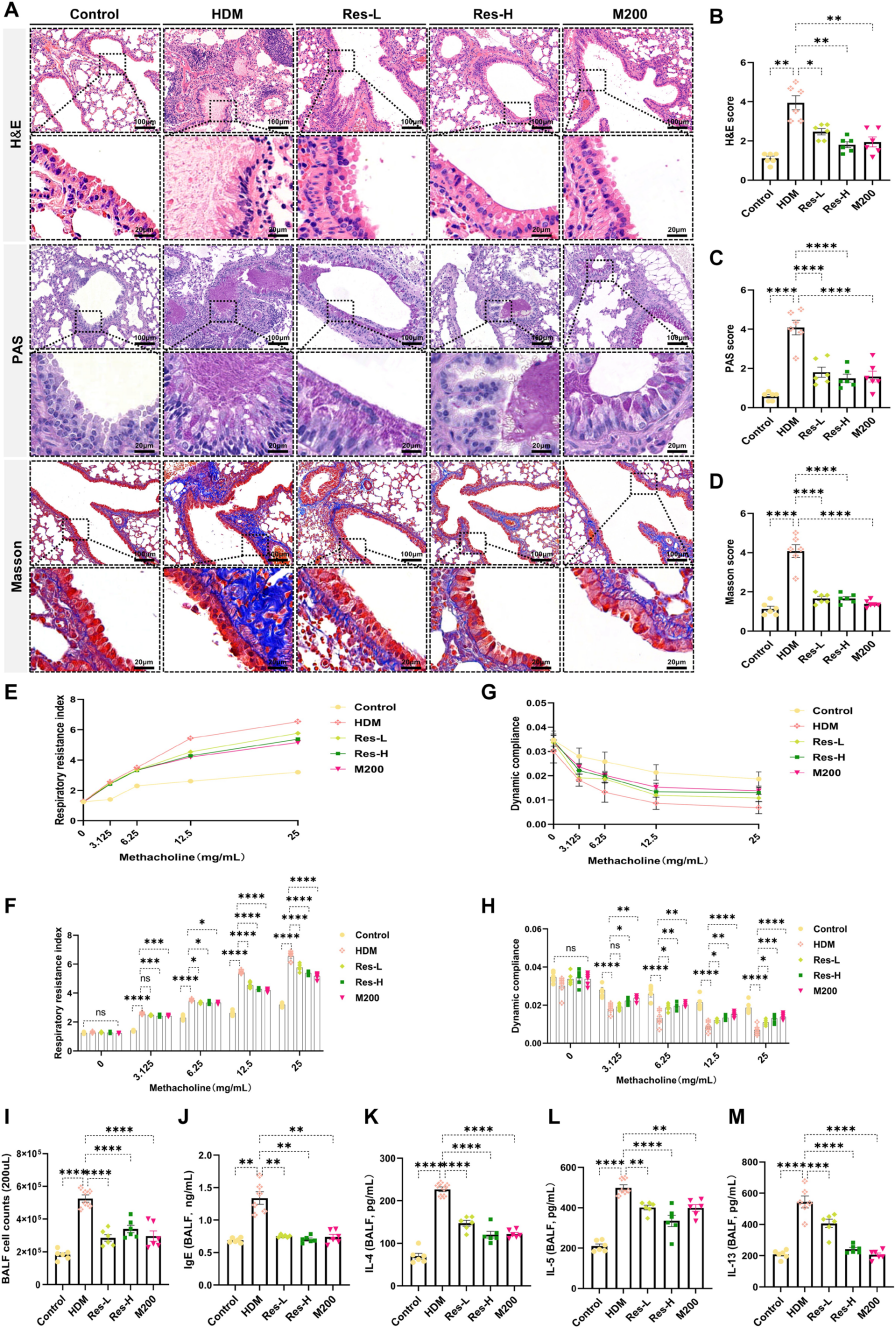
Res and M200 attenuated airway inflammatory responses, structural remodelling, and airway function in asthma mice. (A–D) H&E, PAS, and Masson's trichrome staining were performed to evaluate the effects of Res and M200 on peribronchial inflammatory infiltration, mucus secretion, and fibrosis in lung tissues from asthma mice. *n* = 6 for each group. Scale bars: 100 and 20 μm. (E–H) Airway resistance and dynamic lung compliance were assessed using a Buxco pulmonary function system after methacholine challenge in asthma mice. *n* = 6 for each group. (I) Total inflammatory cell counts in BALF. *n* = 6 for each group. (J–M) ELISA was used to determine the levels of total IgE and inflammatory cytokines IL‐4, IL‐5, and IL‐13 in BALF from asthma mice. *n* = 6 for each group. Data are presented as mean ± SEM. *p < 0.05, **p < 0.01, ***p < 0.001, ****p < 0.0001.

The pharmacological inhibitory effects of Res and M200 on ITGA5 were further evaluated to assess their potential in alleviating airway remodelling and pathological structural changes. Histological analysis of lung tissue sections revealed that Res and M200 reduced peribronchial inflammatory infiltration, airway epithelial cell disorganisation and hyperplasia, basement membrane thickening, excessive mucus secretion, collagen deposition and subepithelial fibrosis in asthma mice (Figure 10A–D). Furthermore, Res and M200 decreased airway resistance, restored lung compliance and improved airway function in HDM‐sensitised asthma mice (Figure 10E–H). Consistently, both agents significantly reduced the total inflammatory cell count in BALF of asthma mice (Figure 10I), as well as the levels of IgE and the secretion of Th2‐type cytokines, including IL‐4, IL‐5 and IL‐13 (Figure 10J–M).

## 4 Discussion

EMT is one of the main causes of airway remodelling and difficulty in controlling asthma. Unfortunately, current insights into the EMT pathogenesis remain fragmented and limited, and existing pharmacological interventions are still inadequate for effectively alleviating asthma airway remodelling. It is necessary to further explore the key targets that affect EMT and potential drug treatments. Although emerging biological agents have shown certain efficacy, their long‐term biosafety still requires more thorough clinical verification, and their expensive cost prevents widespread accessibility [45]. Therefore, it is now essential to develop more feasible and affordable alternative treatment approaches. This study established a link between ITGA5 and anoikis resistance for the first time, and systematically elucidated the critical regulatory role of ITGA5 in TGF‐*β*1‐driven EMT in bronchial epithelial cells as well as airway remodelling in HDM‐sensitised asthma mouse models. Mechanistically, ITGA5 may exacerbate airway structural changes in asthma by coordinating EMT processes and anoikis resistance via activating the PI3K/Akt pathway.

We observed significant elevation of ITGA5 in both asthma patients and bronchial epithelial cells undergoing TGF‐*β*1‐induced EMT in vitro. Moreover, ITGA5 expression levels correlated strongly with impaired lung function, asthma severity, and classic EMT markers, including Fibronectin, N‐cadherin and Vimentin. This suggests that elevated ITGA5 may predict more advanced airway remodelling and impairment. Beyond its traditional integrin partners, ITGA5 interacted extensively with multiple EMT regulators according to subsequent ITGA5 protein interaction network research. This indicates that ITGA5 contributes to EMT through a complex network rather than a single pathway. EMT is characterised by the loss of intercellular adhesion and apicobasal polarity in epithelial cells, cytoskeletal reorganisation, acquisition of a motile, invasive mesenchymal phenotype, and enhanced resistance to apoptosis [5, 6]. ITGA5 was found to improve integrin‐mediated cell‐matrix adhesion and downstream signalling upon binding to Fibronectin, positioning ITGA5 as a potent EMT driver [46, 47]. Moreover, increased DEPs implicated in EMT pathogenesis were found by quantitative proteomic analysis; these DEPs were primarily enriched for molecular functions of integrin binding and ECM protein binding. These DEPs had a major effect on the PI3K/Akt signalling pathway and were associated with biological processes including negative regulation of anoikis and integrin biosynthetic. Notably, of particular interest, this study provided the first observation of anoikis resistance in TGF‐*β*1‐induced EMT of bronchial epithelial cells, offering preliminary evidence for the dual roles of ITGA5 in controlling adhesion‐mediated signalling and cell survival during airway remodelling.

Although anoikis resistance has been extensively documented in tumour‐derived EMT models, its biological significance in non‐neoplastic airway epithelial EMT, such as during airway remodelling or fibrosis, is still completely unknown [48]. Anoikis resistance is essential for EMT‐associated cell migration and metastasis because it allows cells to live and multiply after separating from the extracellular matrix [23]. PI3K/Akt signalling is closely linked to integrin signalling as a crucial route controlling cell motility, invasion and proliferation. Remarkably, ITGA5 participates both in the transduction of PI3K/Akt signalling and the negative regulation of anoikis. In oncogenic cells, ITGA5 has been shown to drive EMT via the PI3K/Akt/mTORC1 pathway [49]. In lung cancer, PI3K/Akt activation promotes EMT while suppressing caspase activity, conferring anoikis resistance to lung cancer cells [50, 51, 52]. Given the dual roles of ITGA5 in integrin signalling and anoikis regulation in cancer models, we hypothesised that ITGA5 facilitates EMT progression by activating PI3K/Akt while concurrently promoting anoikis resistance, suggesting its potential as a predictive biomarker for anoikis resistance. Building on these insights, we next explored whether targeted inhibition of ITGA5 could restore epithelial homeostasis and stop remodelling in vitro. Functional validation experiments revealed that knockdown of ITGA5 expression in bronchial epithelial cells could effectively reverse TGF‐*β*1‐induced EMT, suppress PI3K/Akt pathway activation, decrease anti‐apoptotic proteins XIAP and MCL‐1, as well as anoikis resistance marker TrkB, and increase apoptotic proteins Caspase 9, Cleaved‐caspase 9, Caspase 3 and Cleaved‐caspase 3, activating the mitochondrial intrinsic apoptotic pathway and restore anoikis sensitivity. Furthermore, fluorescence co‐localisation assays demonstrated co‐expression and upregulation of ITGA5, PI3K and TRKB in the bronchial epithelial cells, providing direct cytological evidence in support of our hypothesis.

According to available data, cells that undergo a complete EMT may avoid mitochondrial apoptosis by activating the PI3K/Akt survival cascade or upregulating anti‐apoptotic proteins like MCL‐1 [53] This would counteract pro‐apoptotic cues and lessen apoptotic sensitivity. TGF‐*β* quickly activates PI3K, which phosphorylates its downstream effector Akt. This inhibits Smad‐mediated transcription and shields cells from TGF‐*β*‐induced growth inhibition and apoptosis [54]. During EMT, loss of epithelial adhesion proteins like E‐cadherin combined with increased expression of mesenchymal markers (N‐cadherin, Fibronectin and Vimentin) enables cells to survive in an anchorage‐independent state without cell–cell adhesion [55, 56]. These changes facilitate cell survival in anchorage‐independent states, ensuring the persistence and functionality of EMT cells and driving disease progression in cancers and pulmonary fibrosis [57]. TrkB, a receptor tyrosine kinase, confers anoikis resistance by activating the PI3K/Akt signalling cascade, thereby inhibiting caspase‐3 activation and enhancing post‐detachment cell survival [58]. Moreover, TrkB overexpression robustly induces an EMT‐like phenotype in epithelial cells, characterised by upregulation of mesenchymal markers and concurrent induction of the EMT transcription factors twist and snail [59].

Chronic obstructive pulmonary disease patients exhibiting an airway epithelial anoikis resistance subphenotype trend to present with more advanced disease stages [60]. In idiopathic pulmonary fibrosis (IPF), anoikis resistance has been shown to drive fibroblast activation and ECM deposition, whereas ITGA5 knockdown in IPF‐derived human lung fibroblasts reduces cell proliferation and migration while increasing apoptosis [22]. In asthma airway epithelia, Th2 cytokines and other mediators induce upregulation of inhibitors‐of‐apoptosis (IAP) family proteins [61, 62, 63], correlating with decreased epithelial cell apoptosis and enhanced survival. These changes, in turn, contribute to epithelial thickening, goblet cell hyperplasia, and mucus hypersecretion, thereby exacerbating airway remodelling and chronic inflammation [64]. Furthermore, TGF‐*β*1 has been demonstrated to confer anti‐apoptotic protection in both the 16HBE14o cell line and primary human bronchial epithelial cells by inhibiting Fas‐mediated, Smad‐dependent apoptotic signalling [65]. Based on these findings, we hypothesise that asthma patients with airway remodelling sharing pathological features of ECM remodelling may concurrently exhibit anoikis resistance in airway epithelia. Putatively, airway epithelial cells that undergo EMT and acquire anoikis resistance in asthma might persist in the airway microenvironment as transformed mesenchymal cells, evading normal clearance mechanisms and promoting the chronic and progressive nature of airway remodelling rather than facilitating effective tissue repair and lesion resolution.

Given the key role of ITGA5 in EMT of asthma airway epithelium and its association with the PI3K/Akt pathway and anoikis resistance, we further explored the pharmacological potential of drugs targeting ITGA5. Our findings demonstrated that Res and M200 effectively inhibited ITGA5 expression, in both TGF‐*β*1‐induced in vitro EMT models and HDM‐sensitised asthma mouse models, attenuated EMT phenotypes, blocked the activation of the PI3K/Akt pathway, and restored cellular sensitivity to anoikis. Ultimately, these interventions alleviated airway inflammatory infiltration, epithelial cell disorganised proliferation, basement membrane thickening, mucus hypersecretion and collagen fibre deposition in asthma mice; reduced pulmonary allergic markers such as IgE and the secretion of pulmonary inflammatory cytokines (IL‐4, IL‐5 and IL‐13); and effectively mitigated airway resistance in asthma mice, restoring pulmonary dynamic compliance, thereby improving asthma airway remodelling. Res, a naturally occurring polyphenolic compound, has been shown to downregulate ITGA5 at both the mRNA and protein levels, suggesting its potential as a pharmacological inhibitor of ITGA5 [66]. It has also been identified as a potent EMT‐reversing agent capable of upregulating E‐cadherin while reducing the expression of N‐cadherin and Vimentin. These effects are accompanied by enhanced epithelial tight junction integrity, suppression of PI3K/Akt signalling [67, 68], and inhibition of cell proliferation, invasion, migration and resistance to apoptosis, particularly under TGF‐*β*1‐induced EMT conditions [69]. In bronchial epithelial cells (BEAS‐2B), Res has been shown to downregulate mesenchymal markers such as Vimentin and *α*‐SMA and inhibit the expression of key EMT transcription factors, including Snail1 and Slug. Furthermore, in an ovalbumin‐induced murine model of asthma, Res improved airway hyperresponsiveness, airway inflammation and structural remodelling, which were manifested as goblet cell hyperplasia and collagen deposition [70]. M200, a humanised monoclonal antibody targeting integrin *α*5*β*1, binds specifically to this integrin and disrupts its interaction with extracellular matrix ligands, such as Fibronectin [71]. This blockade interferes with ITGA5‐mediated cell adhesion and downstream signalling. Notably, pharmacological inhibition of ITGA5 by M200 can recapitulate the inhibitory effects observed following ITGA5 knockdown in tumour cell models [72]. Although previous studies have not explicitly demonstrated that M200 modulates epithelial or mesenchymal marker expression, its ability to inhibit ITGA5 suggests a plausible therapeutic role in mitigating EMT. Preclinical data have consistently shown that M200 suppresses proliferation and tube formation of human umbilical vein endothelial cells in vitro and selectively induces apoptosis in proliferating endothelial cells [73]. In vivo, M200 exhibits strong anti‐angiogenic and anti‐tumour activities across various disease models [73]. Importantly, the present study provides direct evidence that M200 can ameliorate EMT in airway epithelial cells and preliminarily reveals its role in promoting apoptosis, supporting its potential application in reversing EMT and overcoming anoikis resistance.

This study significantly advanced our understanding of the role of ITGA5 in EMT of asthma airway epithelium and its underlying mechanism; there are still some limitations. First, despite utilising public database data from asthma patient samples, the causal relationship and long‐term impact of ITGA5 on airway remodelling in asthma patients remain to be validated by large‐scale prospective clinical studies. Second, although the evidence obtained from in vitro experiments and the HDM‐sensitised mouse model of asthma in this study laid a foundation for subsequent clinical translation, further validation in humanised mouse models that faithfully recapitulated the human immune system and mimicked asthma airway inflammation and remodelling would have offered more clinically relevant insights [74, 75, 76]. Such models could have better evaluated the specific mechanisms by which ITGA5 contributed to airway remodelling in asthma patients, as well as the therapeutic efficacy of ITGA5‐targeted inhibitors. Finally, the precise regulatory interactions between EMT and anoikis resistance in the airway epithelium during asthma progression warrant deeper mechanistic investigation.

## 5 Conclusions

In conclusion, this study is the first to identify a critical regulatory role for ITGA5 in promoting EMT in asthma airways and to clarify its potential mechanism of affecting EMT and anoikis resistance through the PI3K/Akt pathway. More importantly, this study predicted and verified the potential of Res and M200 to pharmacologically inhibit ITGA5 to alleviate EMT and anoikis resistance. These findings provide novel insights into the pathophysiology of EMT in asthma and highlight ITGA5 as a potential predictive biomarker and therapeutic target and offer a promising pharmacological strategy for the development of new treatments aimed at mitigating airway remodelling in asthma.

## Author Contributions

**Ting Wang:** experimental design, validation, data analysis, visualisation, writing – original draft. **Ling Rao:** data curation, formal analysis, writing‐review and editing. **Xiaofang Li:** data curation, validation. **Miaofen Zhang:** methodology, data curation, validation. **Huiting Huang:** review and editing, polishing. **Zhiyan Luo:** review and editing, polishing. **Gang Liao:** review and editing, polishing. **Yong Jiang:** review and editing, polishing, funding acquisition. **Shaofeng Zhan:** conceptualization, funding acquisition, review. **Qiong Liu:** supervision, review, polishing. **Xiufang Huang:** conceptualization, data curation, investigation, project administration, funding acquisition, supervision, polishing.

## Funding

This work was supported by the National Natural Science Foundation of China (grant no. 82204985); Natural Science Foundation of Guangdong Province, China (grant no. 2023A1515010807, 2024A1515012183); “Jie bang gua shuai” Special Program for Postgraduate Innovative Ability Enhancement of the First Clinical Medical College (grant no. A3‐0317‐25‐110‐001); Chinese Association of Chinese Medicine (CACM) Young Talent Support Program (grant no. CACM‐2024‐QNRC2‐B38); “Guben” Project for First‐Class Discipline Capacity Improvement of Guangzhou University of Chinese Medicine (grant no. GZY2025GB0112); Sanming Project of Medicine in Shenzhen (grant no. SZZYSM202206013); National Chinese Medicine Advantageous Specialty Construction Project (Pulmonary Disease Department of the First Affiliated Hospital of Guangzhou University of Chinese Medicine); Seventh Batch of “Guangdong Special Support Program” Provincial Health and Health Commission (Health and Health Talents) Project (Grant no. 0720240224); Guangzhou University of Chinese Medicine Young Top Talents (Team) Cultivation “Unveiling the List of Commanders” Project and Guangdong Province Key Departments (Chinese and Western Medicine Collaborative Departments) Construction Project; Guangdong Provincial Bureau of Traditional Chinese Medicine Project, (grant no. 20254043, 20251334, 20241265, 20231296).

## Ethics Statement

All animal procedures were reviewed and approved by the Ethics Committee of the Animal Experiment Center at Guangzhou University of Chinese Medicine (Approval number: 20251023007).

## Conflicts of Interest

The authors declare no conflicts of interest.

## Supporting information


**Figure S1:** (A) Prediction of drugs targeting ITGA5. (B) Docking affinity scores of Res and ITGA5 at different sites, a score < −4.25 kcal/mol indicates good docking affinity; a score < −7 kcal/mol is considered to have strong docking affinity.
**Figure S2:** (A) Lentiviral vector–mediated delivery of distinct ITGA5 shRNA target sequences. (B) Transduction efficiency of each ITGA5‐targeting shRNA in HBE135‐E6E7 cells; scale bar = 500 μm. (C–D) Efficiency of ITGA5 knockdown by different shRNA sequences, as assessed by Western blot (*n* = 3). *n* = 3 for each group. *p < 0.05, **p < 0.01, ***p < 0.001, ****p < 0.0001.

## Data Availability

The data that support the findings of this study are available from the corresponding author upon reasonable request.
