# Supplementary material for: ITGA5 as a Dual Regulator of Epithelial‐Mesenchymal Transition and Epithelial Cell Anoikis Resistance: Functional Validation and Drug Prediction
Source: Cell Prolif. 2026 Mar 2;59(7):e70190. doi: 10.1111/cpr.70190 (PMC13325474; doi:10.1111/cpr.70190)

**Figure S1
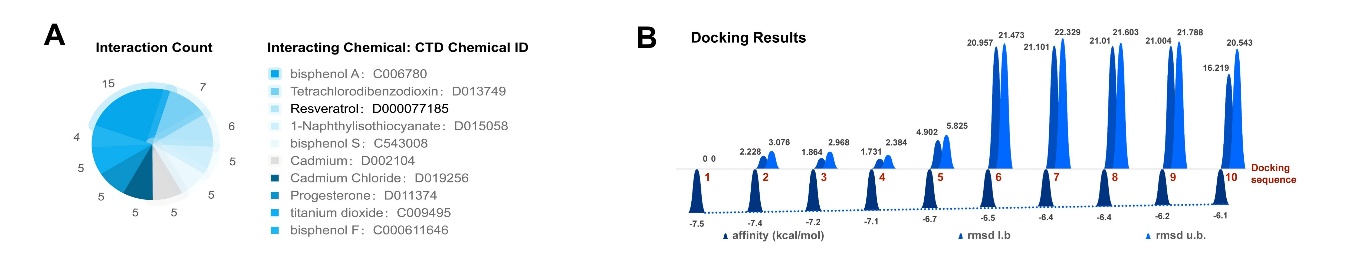
**

**(A)** Prediction of drugs targeting ITGA5. **(B)** Docking affinity scores of Res and ITGA5 at different sites, a score < -4.25 kcal/mol indicates good docking affinity; a score < -7 kcal/mol is considered to have strong docking affinity.

**Figure S2**

**(A)** Lentiviral vector–mediated delivery of distinct ITGA5 shRNA target sequences. **(B)** Transduction efficiency of each ITGA5-targeting shRNA in HBE135-E6E7 cells; scale bar = 500 μm. **(C–D)** Efficiency of ITGA5 knockdown by different shRNA sequences, as assessed by Western blot (n = 3). n=3 for each group. **p* < 0.05, ***p* < 0.01, ****p* < 0.001, *****p* < 0.0001.
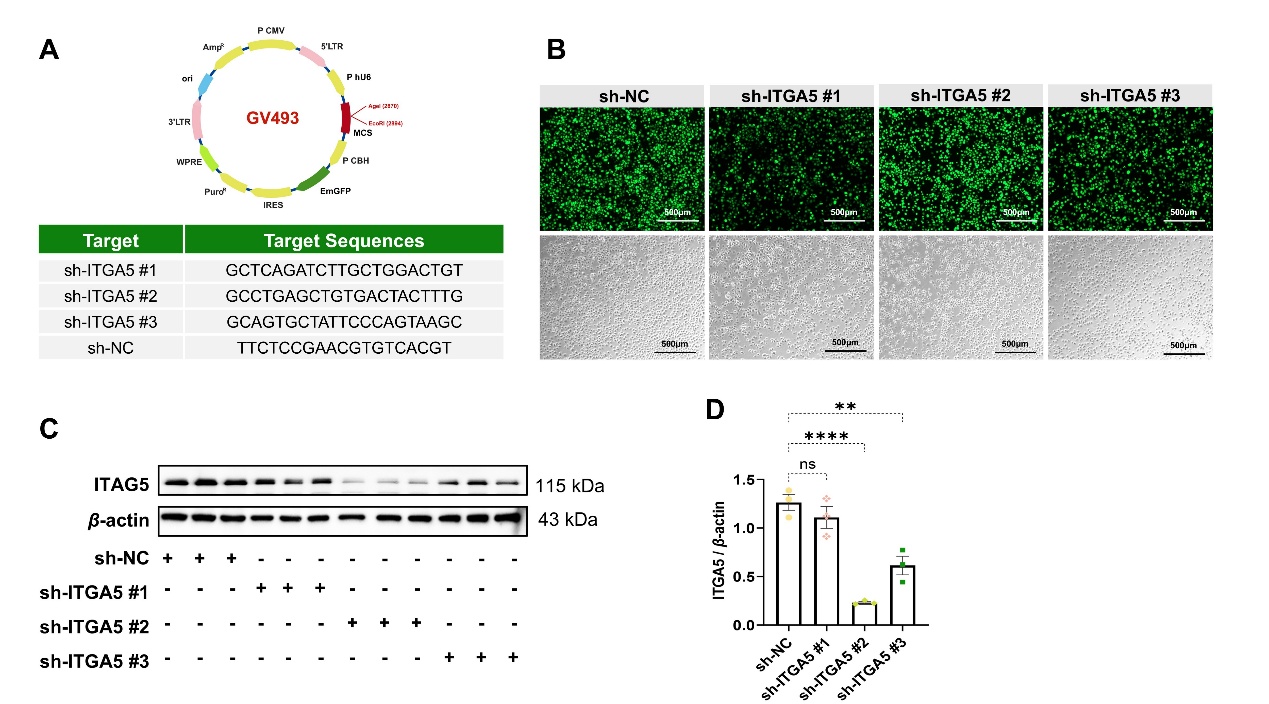

Supplement: Supplementary file 1 — Figure S1: (A) Prediction of drugs targeting ITGA5. (B) Docking affinity scores of Res and ITGA5 at different sites, a score < −4.25 kcal/mol indicates good docking affinity; a score < −7 kcal/mol is considered to have strong docking affinity. Figure S2: (A) Lentiviral vector–mediated delivery of distinct ITGA5 shRNA target sequences. (B) Transduction efficiency of each ITGA5‐targeting shRNA in HBE135‐E6E7 cells; scale bar = 500 μm. (C–D) Efficiency of ITGA5 knockdown by different shRNA sequences, as assessed by Western blot (n = 3). n = 3 for each group. *p < 0.05, **p < 0.01, ***p < 0.001, ****p < 0.0001. [file CPR-59-e70190-s001.docx]
